# Global, regional, and national disease burden estimates of acute lower respiratory infections due to respiratory syncytial virus in children younger than 5 years in 2019: a systematic analysis

**DOI:** 10.1016/S0140-6736(22)00478-0

**Published:** 2022-05-19

**Authors:** You Li, Xin Wang, Dianna M Blau, Mauricio T Caballero, Daniel R Feikin, Christopher J Gill, Shabir A Madhi, Saad B Omer, Eric A F Simões, Harry Campbell, Ana Bermejo Pariente, Darmaa Bardach, Quique Bassat, Jean-Sebastien Casalegno, Giorgi Chakhunashvili, Nigel Crawford, Daria Danilenko, Lien Anh Ha Do, Marcela Echavarria, Angela Gentile, Aubree Gordon, Terho Heikkinen, Q Sue Huang, Sophie Jullien, Anand Krishnan, Eduardo Luis Lopez, Joško Markić, Ainara Mira-Iglesias, Hannah C Moore, Jocelyn Moyes, Lawrence Mwananyanda, D James Nokes, Faseeha Noordeen, Evangeline Obodai, Nandhini Palani, Candice Romero, Vahid Salimi, Ashish Satav, Euri Seo, Zakhar Shchomak, Rosalyn Singleton, Kirill Stolyarov, Sonia KStoszek, Anne von Gottberg, Danielle Wurzel, Lay-Myint Yoshida, Chee Fu Yung, Heather J Zar, Respiratory Virus Global Epidemiology Network investigators, You Li, Xin Wang, Harish Nair, Harry Campbell, Ana Bermejo Pariente, Dianna M Blau, Mauricio T Caballero, Fernando P Polack, Daniel R Feikin, Christopher J Gill, Lawrence Mwananyanda, Shabir A Madhi, Saad B Omer, Eric AF Simões, Rowena Crow, Darmaa Bardach, Tuya Mungun, Naranzul Tsedenbal, Quique Bassat, Sophie Jullien, Jean-Sebastien Casalegno, Yves Gillet, Come Horvat, Etienne Javouhey, Bruno Lina, Dominique Ploin, Giorgi Chakhunashvili, Irakli Karseladze, Nigel Crawford, Danielle Wurzel, Anna-Maria Costa, Andrew Daley, Gregory Walker, Daria Danilenko, Kirill Stolyarov, Lien Anh Ha Do, Annette Alafaci, Alissa McMinn, Kim Mulholland, Claire von Mollendorf, Marcela Echavarria, Noelia Reyes, Angela Gentile, Florencia Lucion, Aubree Gordon, John Kubale, Terho Heikkinen, Q Sue Huang, Claire Newbern, Namrata Prasad, Anand Krishnan, Rakesh Kumar, Eduardo Luis Lopez, Fausto Martín Ferolla, Joško Markić, Ena Batinović, Petra Milić, Ainara Mira-Iglesias, Javier Díez-Domingo, Joan Puig-Barberà, Hannah C Moore, Christopher C Blyth, Amanuel Gebremedhin, Mark P Nicol, Jocelyn Moyes, Anne von Gottberg, Jinal Bhiman, Cheryl Cohen, Mignon du Plessis, Sibongile Walaza, D James Nokes, Nickson Murunga, Faseeha Noordeen, Maduja Divarathna, Evangeline Obodai, John Kofi Odoom, Nandhini Palani, Sujatha Sistla, Candice Romero, Vahid Salimi, Forough Tavakoli, Ashish Satav, Shilpa Satao, Euri Seo, Zakhar Shchomak, Teresa Bandeira, Rosalyn Singleton, Sonia K Stoszek, Rachel Cohen, Lay-Myint Yoshida, Michiko Toizumi, Chee Fu Yung, Heather J Zar, Rae MacGinty, Angel Balmaseda, Ian Barr, Sara S Bressler, Duc-Anh Dang, Christine Desnoyers, Roger Hernandez, Giselle Soto, Adrian Morel, Janine Reiche, Harish Nair, RESCEU investigators, You Li, Xin Wang, Harish Nair, Harry Campbell, Madeleine Edgoose, Yanran Song, Ting Shi, Philippe Beutels, Louis Bont, Andrew J Pollard, Matthew Snape, Peter Openshaw, Federico Martinon-Torres, Terho Heikkinen, Adam Meijer, Thea K Fischer, Maarten van den Berge, Carlo Giaquinto, Michael Abram, Bishoy Rizkalla, Clarisse Demont, Charlotte Vernhes, Jeroen Aerssens

**Affiliations:** School of Public Health, Nanjing Medical University, Nanjing, China; School of Public Health, Nanjing Medical University, Nanjing, China; Center for Global Health, Centers for Disease Control and Prevention, Atlanta, GA, USA; Fundacion INFANT, Buenos Aires, Argentina; Department of Immunizations, Vaccines, and Biologicals, WHO, Geneva, Switzerland; Boston University School of Public Health, Department of Global Health, Boston, Massachusetts, USA; South African Medical Research Council Vaccines and Infectious Diseases Analytics Research Unit; University of the Witwatersrand, Faculty of Health Sciences, Johannesburg, South Africa; Yale Institute for Global Health, New Haven, CT, USA; Department of Pediatrics, Section of Infectious Diseases, University of Colorado, School of Medicine, Aurora, CO, USA; Centre for Global Health, Usher Institute, University of Edinburgh, Edinburgh, UK; Centre for Global Health, Usher Institute, University of Edinburgh, Edinburgh, UK; National Center for Communicable Diseases (Mongolia), Ulaanbaatar, Mongolia; ISGlobal, Hospital Clínic – Universitat de Barcelona, Barcelona, Spain; National Center for Disease Control and Public Health, Tbilisi, Georgia; The Royal Children’s Hospital, Melbourne, Australia; The University of Melbourne, Melbourne, Australia; Smorodintsev Research Institute of Influenza, Saint Petersburg, Russia; Department of Paediatrics; Clinical Virology Unit, Centro de Educación Médica e Investigaciones Clínicas, Buenos Aires, Argentina; Ricardo Gutiérrez Children Hospital, Buenos Aires, Argentina; Department of Epidemiology, University of Michigan, Ann Arbor, MI, USA; Pediatrics, University of Turku and Turku University Hospital, Turku, Finland; WHO National Influenza Centre, Institute of Environmental Science and Research, Wellington, New Zealand; Jigme Dorji Wangchuck National Referral Hospital, Gongphel Lam, Thimphu, Bhutan; Centre for Community Medicine, All India Institute of Medical Sciences, New Delhi, India; Hospital de Niños Dr. Ricardo Gutiérrez, Department of Medicine, Pediatric Infectious Diseases Program, Universidad de Buenos Aires, Buenos Aires, Argentina; Department of Pediatrics, University Hospital Split, Split, Croatia; Área de Investigación en Vacunas, Fundación para el Fomento de la Investigación Sanitaria y Biomédica de la Comunitat Valenciana, Salud Pública, Valencia, Spain; Wesfarmers Centre for Vaccines and Infectious Diseases, Telethon Kids Institute, University of Western Australia, Perth, Australia; National Institute for Communicable Diseases of the National Health Laboratory Service, Johannesburg, South Africa; Boston University School of Public Health, Department of Global Health, Boston, Massachusetts, USA; Kenya Medical Research Institute–Wellcome Trust Research Programme, Kilifi, Kenya; Department of Microbiology, Faculty of Medicine, University of Peradeniya, Peradeniya, Sri Lanka; Virology Department, Noguchi Memorial Institute for Medical Research, University of Ghana, Legon, Accra, Ghana; Department of Microbiology, Jawaharlal Institute of Postgraduate Medical Education & Research, Puducherry, India; Vysnova Partners, Lima, Perú; Department of Virology, School of Public Health, Tehran University of Medical Sciences, Tehran, Iran; MAHAN Trust Mahatma Gandhi Tribal Hospital, Karmgram, Utavali, Tahsil, Dharni, India; Department of Pediatrics, Dongguk University Ilsan Hospital, Dongguk University College of Medicine, Goyang, South Korea; Department of Pediatrics, Hospital Santa Maria, Centro Hospitalar Universitário Lisboa Norte, Lisbon, Portugal; Alaska Native Tribal Health Consortium, Anchorage, AK, USA; The University of Melbourne, Melbourne, Australia; Smorodintsev Research Institute of Influenza, Saint Petersburg, Russia; Glaxo Smith Kline, Rockville, Maryland, USA; Department of Pathology, Faculty of Health Sciences, University of Cape Town, Cape Town, South Africa; Murdoch Children’s Research Institute, Melbourne, Australia; Department of Pediatric Infectious Diseases, Institute of Tropical Medicine, Nagasaki University, Nagasaki, Japan; Infectious Diseases Service, Department of Paediatrics, KK Women’s and Children’s Hospital, Singapore; Department of Paediatrics and Child Health, and South African Medical Research Council Unit on Child & Adolescent Health, University of Cape Town and Red Cross War Memorial Children’s Hospital, Cape Town, South Africa; Nanjing Medical University Nanjing, China; University of Edinburgh; Centers for Disease Control and Prevention, Atlanta, GA, USA; Fundacion INFANT, Buenos Aires, Argentina; WHO, Geneva, Switzerland; Boston University, Boston, MA, USA; University of the Witwatersrand, Johannesburg, South Africa; Yale University, New Haven, CT, USA; University of Colorado, Aurora, CO, USA; National Center for Communicable Diseases, Ulaanbaatar, Mongolia; Universitat de Barcelona, Barcelona, Spain; Hôpital de la Croix-Rousse, Lyon, France; National Center for Disease Control Public Health, Tbilisi, Georgia; The Royal Children’s Hospital, Melbourne, Australia; Smorodintsev Research Institute of Influenza, St. Petersburg, Russian Federation; Murdoch Children’s Research Institute, Melbourne, Australia; Centro de Educación Médica e Investigaciones Clínicas, Argentina; Ricardo Gutiérrez Children Hospital, Buenos Aires, Argentina; University of Michigan, Ann Arbor, MI, USA; University of Turku Turku University Hospital, Turku, Finland; Institute of Environmental Science Research, Wellington, New Zealand; All India Institute of Medical Sciences, New Delhi, India; Hospital de Niños Dr. Ricardo Gutiérrez, Buenos Aires, Argentina; University Hospital Split, Split, Croatia; Fundación para el Fomento de la Investigación Sanitaria y Biomédica de la Comunitat Valenciana, Salud Pública, Valencia, Spain; University of Western Australia, Australia; National Institute for Communicable Diseases of the National Health Laboratory Service, Johannesburg, South Africa; Kenya Medical Research Institute-WellcomeTrust Research Programme, Kilifi, Kenya; University of Peradeniya, Peradeniya, Sri Lanka; University of Ghana, Legon, Accra, Ghana; Jawaharlal Institute of Postgraduate Medical Education & Research, Puducherry, India; Vysnova Partners Inc., Lima, Perú; Tehran University of Medical Sciences, Tehran, Iran; MAHAN Trust Mahatma Gandhi Tribal Hospital, Karmgram, Utavali, Tahsil, Dharni, India; Dongguk University, Goyang, Republic of Korea; Hospital Santa Maria, Centro Hospitalar Universitário Lisboa Norte, Lisbon, Portugal; Alaska Native Tribal Health Consortium, Anchorage, AK, USA; Glaxo Smith Kline, Rockville, MD, USA; Nagasaki University, Nagasaki, Japan; KK Women’s Children’s Hospital, Singapore; University of Cape Town Red Cross War Memorial Children’s Hospital, Cape Town, South Africa; Ministry of Health, Managua, Nicaragua; World Health Organization Collaborating Centre for Reference Research on Influenza, Melbourne, Australia; Centers for Disease Control Prevention, Anchorage, AK, USA); National Institute of Hygiene and Epidemiology, Hanoi, Vietnam; Yukon Kuskokwim Health Corporation, Bethel, AK, USA; Cayetano Heredia National Hospital, Lima, Perú; US Naval Medical Research Unit No6, Lima, Perú; General Hospital, Kegalle, Sri Lanka; Robert Koch Institute, Berlin, Germany; Nanjing Medical University, Nanjing, China; University of Edinburgh, Edinburgh, UK; University of Antwerp, Antwerpen, Belgium; University Medical Centre Utrecht, the Netherlands; University of Oxford, Oxford, UK; Imperial College London, London, UK; Servicio Galego de Saude, Santiago de Compostela, Spain; University of Turku and Turku University Hospital, Turku, Finland; Institute for Public Health and the Environment, Bilthoven, the Netherlands; Statens Serum Institut, Copenhagen, Denmark; University of Groningen, Groningen, the Netherlands; PENTA Foundation, Padua, Italy; AstraZeneca, Gaithersburg, MD, USA; GlaxoSmithKline, Wavre, Belgium; Sanofi Pasteur, Lyon, France; Janssen, Beerse, Belgium

## Abstract

**Background:**

Respiratory syncytial virus (RSV) is the most common cause of acute lower respiratory infection in young children. We previously estimated that in 2015, 33·1 million episodes of RSV-associated acute lower respiratory infection occurred in children aged 0–60 months, resulting in a total of 118 200 deaths worldwide. Since then, several community surveillance studies have been done to obtain a more precise estimation of RSV associated community deaths. We aimed to update RSV-associated acute lower respiratory infection morbidity and mortality at global, regional, and national levels in children aged 0–60 months for 2019, with focus on overall mortality and narrower infant age groups that are targeted by RSV prophylactics in development.

**Methods:**

In this systematic analysis, we expanded our global RSV disease burden dataset by obtaining new data from an updated search for papers published between Jan 1, 2017, and Dec 31, 2020, from MEDLINE, Embase, Global Health, CINAHL, Web of Science, LILACS, OpenGrey, CNKI, Wanfang, and ChongqingVIP. We also included unpublished data from RSV GEN collaborators. Eligible studies reported data for children aged 0–60 months with RSV as primary infection with acute lower respiratory infection in community settings, or acute lower respiratory infection necessitating hospital admission; reported data for at least 12 consecutive months, except for in-hospital case fatality ratio (CFR) or for where RSV seasonality is well-defined; and reported incidence rate, hospital admission rate, RSV positive proportion in acute lower respiratory infection hospital admission, or in-hospital CFR. Studies were excluded if case definition was not clearly defined or not consistently applied, RSV infection was not laboratory confirmed or based on serology alone, or if the report included fewer than 50 cases of acute lower respiratory infection. We applied a generalised linear mixed-effects model (GLMM) to estimate RSV-associated acute lower respiratory infection incidence, hospital admission, and in-hospital mortality both globally and regionally (by country development status and by World Bank Income Classification) in 2019. We estimated country-level RSV-associated acute lower respiratory infection incidence through a risk-factor based model. We developed new models (through GLMM) that incorporated the latest RSV community mortality data for estimating overall RSV mortality. This review was registered in PROSPERO (CRD42021252400).

**Findings:**

In addition to 317 studies included in our previous review, we identified and included 113 new eligible studies and unpublished data from 51 studies, for a total of 481 studies. We estimated that globally in 2019, there were 33·0 million RSV-associated acute lower respiratory infection episodes (uncertainty range [UR] 25·4–44·6 million), 3·6 million RSV-associated acute lower respiratory infection hospital admissions (2·9–4·6 million), 26 300 RSV-associated acute lower respiratory infection in-hospital deaths (15 100–49 100), and 101 400 RSV-attributable overall deaths (84 500–125 200) in children aged 0–60 months. In infants aged 0–6 months, we estimated that there were 6·6 million RSV-associated acute lower respiratory infection episodes (4·6–9·7 million), 1·4 million RSV-associated acute lower respiratory infection hospital admissions (1·0–2·0 million), 13 300 RSV-associated acute lower respiratory infection inhospital deaths (6800–28 100), and 45700 RSV-attributable overall deaths (38 400–55 900). 2·0% of deaths in children aged 0–60 months (UR 1·6–2·4) and 3·6% of deaths in children aged 28 days to 6 months (3·0–4·4) were attributable to RSV. More than 95% of RSV-associated acute lower respiratory infection episodes and more than 97% of RSV-attributable deaths across all age bands were in low-income and middle-income countries (LMICs).

**Interpretation:**

RSV contributes substantially to morbidity and mortality burden globally in children aged 0–60 months, especially during the first 6 months of life and in LMICs. We highlight the striking overall mortality burden of RSV disease worldwide, with one in every 50 deaths in children aged 0–60 months and one in every 28 deaths in children aged 28 days to 6 months attributable to RSV. For every RSV-associated acute lower respiratory infection in-hospital death, we estimate approximately three more deaths attributable to RSV in the community. RSV passive immunisation programmes targeting protection during the first 6 months of life could have a substantial effect on reducing RSV disease burden, although more data are needed to understand the implications of the potential age-shifts in peak RSV burden to older age when these are implemented.

**Funding:**

EU Innovative Medicines Initiative Respiratory Syncytial Virus Consortium in Europe (RESCEU).

## Introduction

Human respiratory syncytial virus (RSV) is the most common pathogen identified in infants and young children with acute lower respiratory infection.^1,2^ We previously estimated^3^ that in 2015, there were 33·1 million episodes of RSV-associated acute lower respiratory infection, 3·2 million hospital admissions for RSV-associated acute lower respiratory infection, and 59 600 in-hospital RSV-associated acute lower respiratory infection deaths in children younger than 5 years.^3^ We also estimated that overall RSV-associated deaths from acute lower respiratory infection could be as high as 118 200, based on an indirect approach that inflated in-hospital mortality estimates due to the absence of RSV mortality data in community settings at the time of the analysis.^3^ Since 2017, new data on RSV burden in young children have become available, including from several new RSV community surveillance studies initiated to measure RSV mortality in the community (RSV community mortality surveillance studies^4^ and child health and mortality prevention surveillance [CHAMPS]^5^ supported by the Bill & Melinda Gates Foundation). Meanwhile, there have been substantial advances in the development of RSV prophylactic products, with several prophylactic candidates in late-phase clinical development.^6^ A monoclonal antibody with extended half-life, nirsevimab (AstraZeneca and Sanofi), which demonstrated high efficacy among healthy preterm infants in a phase 2b trial,^7^ reduced medically-attended RSV lower respiratory tract infections in healthy late-preterm and term infants in its phase 3 trial (NCT03979313).^8^ Moreover, two maternal vaccine candidates aimed to protect infants through transplacental transfer of vaccine-induced maternal antibodies (RSV MAT [GlaxoSmithKline; NCT04605159] and RSVpreF [Pfizer; NCT04424316]), and one monoclonal antibody (MK-1654 [MSD; NCT04767373]) have initiated recruitment for the phase 3 clinical trials, and are expected to complete in the next 3–5 years.

In this study, we aim to estimate RSV-associated acute lower respiratory infection morbidity and mortality in 2019 at global, regional, and national levels in children aged 0–60 months, with a primary focus on narrower infant age groups that are targeted by RSV prophylactics under development, and on overall mortality.

## Methods

### Definitions

As previously described,^3^ acute lower respiratory infection was defined by setting. For community-level setting (eg, primary care), we used WHO Integrated Management of Childhood Illnesses pneumonia case definitions and replaced the terms “clinical pneumonia” with “ALRI”; for hospital setting, we used physician-confirmed diagnosis of acute lower respiratory infection (pneumonia or bronchiolitis). RSV-associated acute lower respiratory infection was defined as acute lower respiratory infection with laboratory-confirmed RSV infection. RSV-attributable acute lower respiratory infection was defined as acute lower respiratory infection that could be causally attributable to laboratory-confirmed RSV infection. Hypoxaemia was defined as SpO_2_ less than 90% (or <87% if at altitude >2500 metres) in children aged 1–60 months and less than 88% (or <85% if at altitude >2500 metres) for children younger than 1 month. For more detailed definitions see the appendix (p 8).

### Search strategy and selection criteria

We conducted a systematic literature review, updating our previous review.^3^ We searched MEDLINE, Embase, Global Health, CINAHL, Web of Science, LILACS, OpenGrey, CNKI, Wanfang, and ChongqingVIP for studies published between Jan 1, 2017, and Dec 31, 2020, that reported RSV-associated acute lower respiratory infection morbidity and mortality estimates in children aged 0–60 months in 2019 or before, (ie, before the onset of the COVID-19 pandemic). The literature search used the terms (with synonyms and closely related words) “respiratory syncytial virus”, “pneumonia”, “bronchiolitis”, “respiratory tract infections”, “incidence”, “morbidity”, “mortality”, “burden”, and “epidemiology”. For a detailed search strategy, see the appendix (pp 4–7). References cited in retrieved articles were also examined for eligibility. No language restrictions were applied. Two authors (YL and ABP) searched and screened the literature independently. Eligible studies reported data for children aged 0–60 months with RSV as primary infection with acute lower respiratory infection in community settings or acute lower respiratory infection necessitating hospital admission; reported data for at least 12 consecutive months, except for in-hospital case fatality ratio (CFR) or where RSV seasonality is well defined (eg, in temperate regions); and reported incidence rate, hospital admission rate, RSV positive proportion in acute lower respiratory infection hospital admission, or in-hospital CFR.

Studies were excluded if case definition was not clearly defined or not consistently applied, RSV infection was not laboratory confirmed or based on serology alone, or if the report included fewer than 50 cases of acute lower respiratory infection.

Data extraction was done independently by two authors (XW and ABP) using a tailored spreadsheet, with any disagreements arbitrated by YL. The data collection spreadsheet collected study-level information, such as location or country, study period, eligibility criteria, case definition, clinical specimen and diagnostic tests, and the reported morbidity and mortality estimates.

### Unpublished RSV data

Previously, we established the Respiratory Virus Global Epidemiology Network (RSV GEN) to collect unpublished data (including re-analysis of published data), applying common case definitions and approaches to data analysis, yielding unpublished data from over 70 individual study sites.^3^ For this study, we continued to encourage existing members of RSV GEN to contribute new RSV data while inviting other investigators with eligible RSV data to join this collaborative network. This resulted in novel unpublished data from 51 study sites to supplement published data and data included in our previous estimates (appendix pp 9–11).

### Quality assessment

For both published and unpublished RSV data, two authors (YL and XW) conducted quality assessment at the study level independently using a self-designed quality scoring form (appendix p 18).^9–11^ The quality scoring form assessed the study quality and risk of bias on study design, subjects, case definition, sampling strategy (for RSV testing), and diagnostic tests; for hospital-based studies, adjustment for health-care utilisation and ascertainment for hypoxaemia were also assessed where applicable. Based on the individual assessment questions above, an overall quality score was calculated for each study, ranging between 0 (lowest quality) and 1 (highest quality). Regardless of the scores, all studies were included in main analysis; studies with quality scores <0·6 were excluded in sensitivity analysis.

### Data analysis

**Figure 1:**
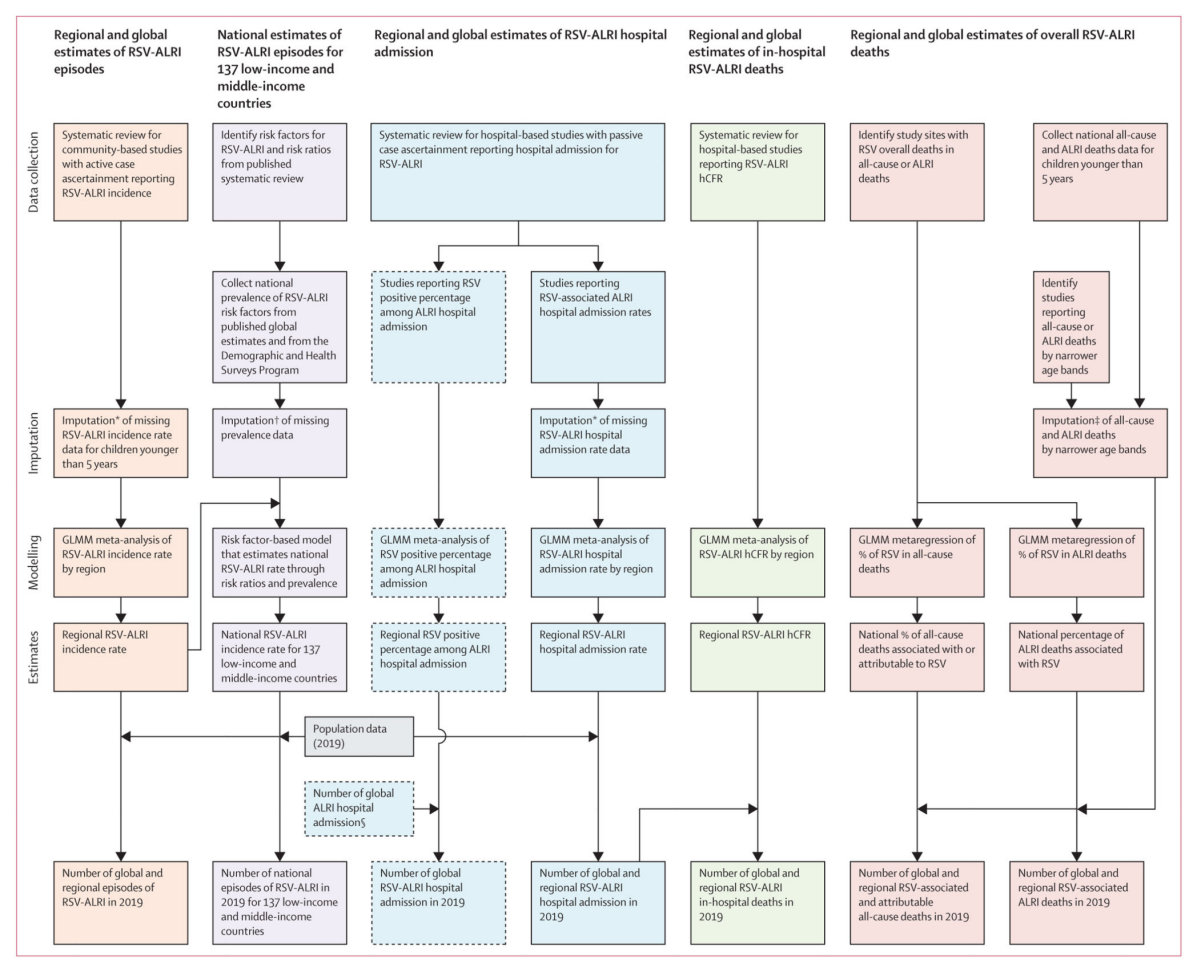
Approaches for estimation of global RSV morbidity and mortality in children aged 0–60 months Boxes with solid borders are main analyses. Boxes with dashed borders are sensitivity analyses. RSV=respiratory syncytial virus. ALRI=acute lower respiratory infection. GLMM=generalised linear mixed-effects model. hCFR= in-hospital case fatality ratio. *Details in the appendix (p 12). †Details in the appendix (p 13). ‡Details in the appendix (p 17). §Details in the appendix (p 19).

We estimated RSV-associated acute lower respiratory infection incidence, RSV-associated acute lower respiratory infection hospital admission, RSV-associated acute lower respiratory infection in-hospital deaths, and RSV-associated and RSV-attributable overall deaths for children aged 0–60 months and, where data were available, for narrower age bands (figure 1).

We conducted meta-analysis of RSV-associated acute lower respiratory infection incidence rate and hospital admission rate by regions (UNICEF country development status and World Bank income classification),^12^ age band, and severity (ie, acute lower respiratory infection with or without chest wall indrawing, and with or without hypoxaemia), through a generalised linear mixed-effects model (GLMM) of two levels (within-study and between-study).^13^ As we prioritised the geographical representativeness of our study sites, we were flexible in terms of study years considered in the meta-analysis. We adjusted for proportion of acute lower respiratory infection cases not tested for RSV by applying the RSV positive proportion among acute lower respiratory infection cases tested for RSV to the total tested and untested acute lower respiratory infection cases, if it was not done already in the individual studies. Data imputation was done for studies not reporting RSV-associated acute lower respiratory infection incidence rate or hospital admission rate for children aged 0–60 months (appendix p 12). The incidence rate and hospital admission rate meta-estimates were applied to regional population estimates for 2019^12^ to yield the number of RSV-associated acute lower respiratory infection episodes and hospital admission for RSV-associated acute lower respiratory infection or RSV-associated acute lower respiratory infection with hypoxaemia.

For RSV-associated acute lower respiratory infection incidence, we also estimated country-specific RSV-associated acute lower respiratory infection incidence rate and episodes in children aged 0–60 months in 137 LMICs using a risk-factor based model similar to previous work (appendix pp 13–14).^3^ For RSV-associated acute lower respiratory infection hospital admission, we validated the estimates with independent data by applying the meta-estimates of RSV positive proportion among acute lower respiratory infection hospital admissions (through GLMM) in our study to the external sources of acute lower respiratory infection hospital admission estimates (appendix p 19 and p 42).^14,15^

We obtained meta-estimates of in-hospital CFR for RSV-associated acute lower respiratory infection hospital admission by region (UNICEF country development status and World Bank Income classification) and age band using GLMM. Then we applied the in-hospital CFR to the RSV-associated acute lower respiratory infection hospital admission estimates, by region and age band, to calculate RSV-associated acute lower respiratory infection in-hospital deaths.

With RSV community mortality data from 2017–19,^16–20^ we developed a new suite of models for estimating RSV-associated and attributable overall deaths (ie, in-hospital and out-of-hospital or community deaths) for children aged 0–60 months and for narrower age bands, both regionally and globally. Rather than inflating indirectly from in-hospital estimates as previously, we aimed to model the RSV proportion among overall all-cause deaths (as the main model) and among overall acute lower respiratory infection deaths (as secondary model). Both models were based on a GLMM framework that accounted for study setting (ie, community only *vs* community and in-hospital), method for RSV confirmation, age band (0–27 days, 28 days–6 months, 6–12 months, and 12–60 months), country’s under-5-years all-cause mortality rate, and method for assigning cause of death (ie, whether only verbal autopsy was available for the secondary acute lower respiratory infection model) as model covariates.

The three recorded outcomes were RSV-attributable deaths (primary outcome, defined as RSV being in the causal chain based on CHAMPS, including RSV being the underlying, intermediate [comorbid or antecedent causes], and immediate causes of death^5,16^), RSV-associated all-cause, and acute lower respiratory infection deaths (secondary outcomes, defined as RSV being tested positive in upper respiratory samples). Detailed methods are presented in the appendix (pp 16–17).

For estimates that were generated from single metaanalysis, the uncertainty range (UR) was derived from the coefficient and its standard error of that metaanalysis. For estimates that were generated through results from multiple meta-analyses, the UR of the estimates were generated using the Monte Carlo simulation to avoid inflation of the UR, based on 1000 samples of each of the meta-estimates from log-normal distributions, with 2·5th percentile and 97·5th percentile defining the lower and upper bounds.^3^

All data analyses were done using R software (version 4.0.5). The study data and R codes for the analysis are available in Edinburgh DataShare (https://doi.org/10.7488/ds/3138). This study was conducted and reported in accordance with the Guidelines for Accurate and Transparent Health Estimates Reporting (GATHER) recommendations (appendix p 51). The systematic literature review was reported in accordance with the Preferred Reporting Items for Systematic Reviews and Meta-Analyses (PRISMA) checklist 2020 (appendix pp 52–54). This review was registered in PROSPERO (CRD42021252400).

### Role of the funding source

The funder had no role in the study design, data collection, data analysis, data interpretation, writing of the report, or the decision to submit.

## Results

In addition to the 317 studies included in the previous review,^3^ we identified and included 113 new eligible studies from the literature review update and included new unpublished data from 51 studies from RSV GEN collaborators. This brought the total number of studies included in the present analysis to 481. Among the 481 studies, 140 contributed to the estimate of RSV-associated acute lower respiratory infection incidence or hospitalisation rate; 339 contributed to the estimate of RSV-positive proportion among acute lower respiratory infection hospital admission; 147 contributed to the estimate of RSV-associated in-hospital deaths; and 15 studies contributed to the estimate of RSV overall deaths (appendix pp 20–23). Study-level characteristics are presented in the appendix (pp 24–31). Through regional-level meta-analyses, we estimated that 33·0 million (UR 25·4–44·6 million) RSV-associated acute lower respiratory infection episodes occurred globally in children aged 0–60 months in 2019, with one in five episodes occurring in infants aged 0–6 months (6·6 million, 4·6–9·7 million). In low-income and lower-middle-income countries, the RSV-associated acute lower respiratory infection incidence rate peaked in children aged 0–3 months whereas the rate peaked in children aged 3–6 months in upper-middle-income and high-income countries. More than 95% of RSV-associated acute lower respiratory infection episodes occurred in low-income and middle-income countries (LMICs) across all age groups; table 1) Country-level estimates from the risk-factor-based model showed substantial variations in RSV-associated acute lower respiratory infection rate among LMICs, ranging from 40·3 per 1000 children per year in China (95% UR 29·7-54·6) to 83·4 per 1000 children per year in Eswatini (61·6–113·1; appendix pp 32–34). Approximately 5·5 million (17%) of 33·0 million RSV-associated acute lower respiratory infection episodes had chest wall indrawing (2·2–15·7 million). Infants aged 0–6 months had the highest incidence of RSV-associated acute lower respiratory infection with chest wall indrawing, with an estimated 2·3 million episodes (1·4–4·3 million), which accounted for 36% of all 6·6 million acute lower respiratory infection episodes in this age group (4·6–9·7 million; table 1).

We estimated that there were 3·6 million RSV-associated acute lower respiratory infection hospital admissions globally in children aged 0–60 months in 2019 (UR 2·9–4·6 million), approximately 1·4 million (39%) of which occurred in infants aged 0–6 months (1·0–2·0 million). There was little variation in admission to hospital for RSV-associated acute lower respiratory infection among different income regions, although this rate was lower in low-income countries. The rate of hospital admission for RSV-associated acute lower respiratory infection peaked in children aged 0–3 months across all regions (more specifically at 28 days to 3 months; appendix pp 36–37). Approximately 0·9 million (26%) of 3·6 million hospital admissions for RSV-associated acute lower respiratory infection had hypoxaemia (UR 0·5–1·9 million). In infants aged 0–6 months, we estimated that there were 0·4 million hospital admissions for RSV-associated acute lower respiratory infection with hypoxaemia (0·2–0·8 million). Within the first six months of life, more than 60% of the RSV-associated acute lower respiratory infection hospital admissions were during the first three months of life (table 2).

The proportion of patients admitted to hospital for acute lower respiratory infection who were positive for RSV was highest in high-income countries for both children aged 0–60 months (29% in high-income countries *vs* 23–26% in LMICs) and those aged 0–6 months (50% for high-income countries *vs* 32–33% in LMICs). Among narrower age bands, the proportion of admitted patients with acute lower respiratory infection peaked in children aged 0–3 months (32–51% across different income levels) and then decreased with increasing age (15–24% in children aged 12–60 months across different income levels; appendix p 38). As a sensitivity analysis, we applied these proportion results to external estimates^14,15^ of hospital admissions for acute lower respiratory infection to cross-validate our estimate for hospital admission for RSV-associated acute lower respiratory infection, which yielded an estimated 1·3–4·1 million hospital admissions for RSV-associated acute lower respiratory infection, consistent with our primary point estimate of 3·6 million (appendix p 39).

We estimated that the in-hospital CFR of RSV-associated acute lower respiratory infection ranged from 0·1% (UR 0·1–0·2) in high-income countries to 1·4% (0·6–2·8) in low-income countries among children aged 0–60 months. The regional variation in in-hospital CFR was even more pronounced in children aged 0–6 months. Although the in-hospital CFR remained consistently low in high-income countries across all age groups, there were substantial variations in in-hospital CFR across age groups in developing countries; for example, CFR was highest in children aged 0–3 months (1·1%, UR 0·7–1·8) and lowest in children aged 12–60 months (0·5%, 0·3–1·0) in developing countries. Our stratified analysis by the median study year (which was 2012) suggests that there was substantial decrease in in-hospital CFR over time among developing countries (0·99%, 0·69-1·45 before 2012 *vs* 0·54%, 0·31-0·98 after 2012 in children aged 0–60 months) but limited decrease was observed in industrialised countries (0·11%, 0·07–0·16 *vs* 0·08%, 0·02–0·31 in children aged 0–60 months; appendix p 41). At the global level, in 2019, we estimated that there were 26 300 (UR 15 100–49 100) RSV-associated acute lower respiratory infection in-hospital deaths in children aged 0–60 months, 13 300 (51%, 6800–28 100) of which occurred in children aged 0–6 months. LMICs accounted for more than 97% of RSV-associated acute lower respiratory infection in-hospital deaths across all age groups in children younger than 60 months (table 3).

To further supplement these RSV-associated morbidity and in-hospital mortality estimates, we also estimated the acute lower respiratory infection morbidity and in-hospital mortality that could be attributable to RSV (appendix p 15). We estimated that in 2019, there were 29·7 million (22·8–40·2 million) acute lower respiratory infection episodes, 3·2 million (2·6–4·2 million) hospital admissions for acute lower respiratory infection, and 21 100 (12 100–39 300) in-hospital deaths for acute lower respiratory infection that could be attributable to RSV in children aged 0–60 months.

**Figure 2:**
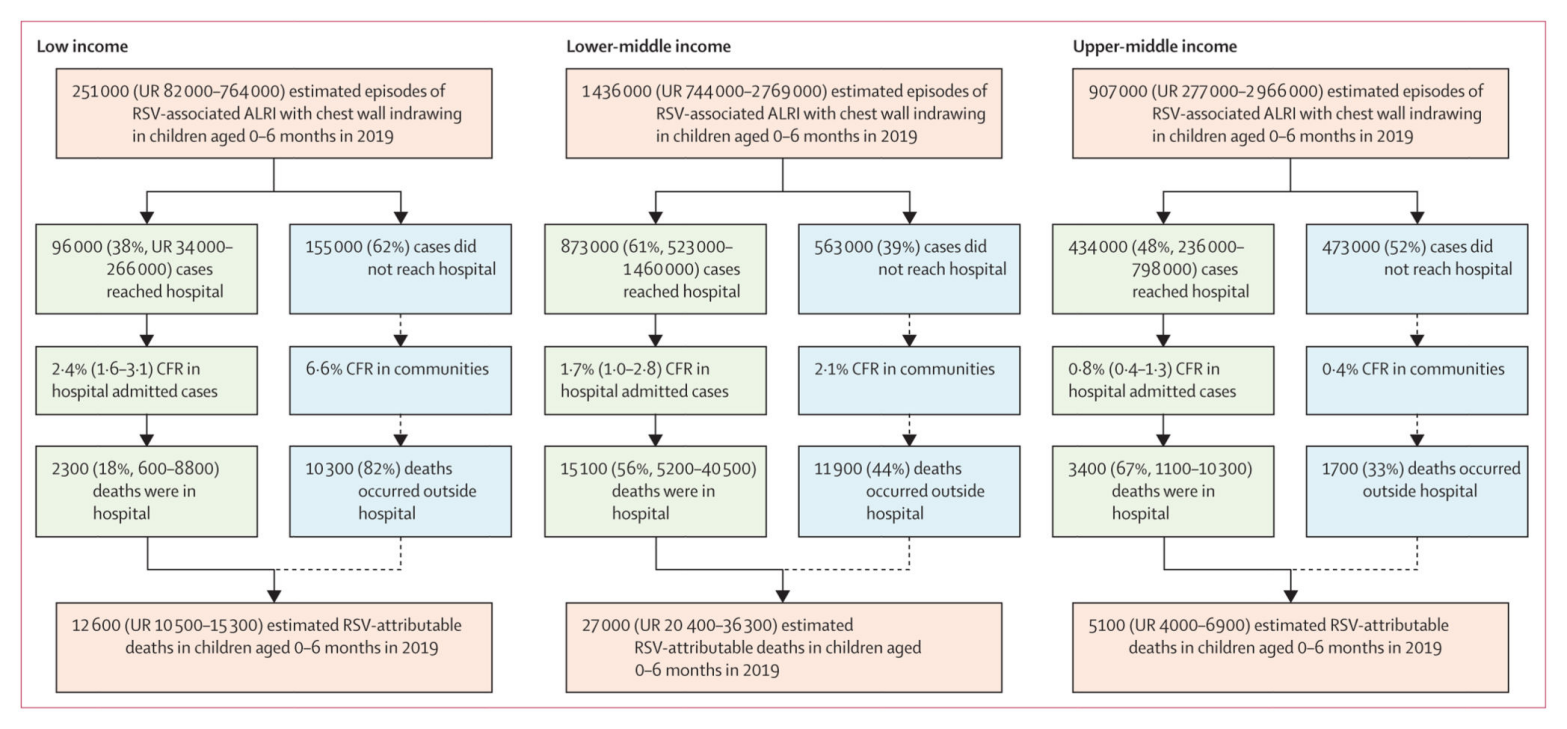
Burden of RSV-associated ALRI in infants aged 0–6 months in LMICs by severity and outcome including burden on health-care services ALRI=acute lower respiratory infection. CFR=case fatality ratio. LMICs=low and middle income countries. RSV=respiratory syncytial virus. UR=uncertainty range.

For overall RSV deaths, we estimated that 101 400 (2·0%, UR 84 500-125 200) of 52 million all-cause deaths were attributable to RSV in children aged 0–60 months and this proportion was highest in children aged 28 days-6 months (3·6%, 3·0–4·4). Although only 0·7% of all-cause deaths during the neonatal period (age 0–27 days) were attributable to RSV, RSV deaths inneonates still accounted for 19% of the RSV-attributable deaths in the first six months of life. LMICs accounted for more than 97% of the RSV-attributable deaths across all age groups. As a secondary estimate, we computed that there were 229 000 (UR 196 000–271 200) RSV-associated all-cause deaths and 109 600 (97 200-124 900) RSV-associated acute lower respiratory infection deaths in children aged 0–60 months in 2019 (table 4). Taken with the estimated RSV-associated acute lower respiratory infection in-hospital deaths, only 26 300 (26%) of 101 400 RSV-attributable deaths occurred in hospitals in patients aged 0–60 months and 13 300 (29%) of 45 700 RSV-attributable deaths occurred in hospitals in children aged 0–6 months. Only 2300 (18%) of 12 500 RSV-attributable deaths occurred in hospitals in infants aged 0–6 months in low-income countries, and CFR was as high as 6·6% in communities (figure 2).

## Discussion

In this study, we expanded our existing global RSV disease burden dataset by including 113 new eligible studies from the literature review update and new unpublished data from 51 studies shared through RSV GEN. We estimated that globally in 2019, there were 33·0 million RSV-associated acute lower respiratory infection episodes, 3·6 million RSV-associated acute lower respiratory infection hospital admissions, and 26 300 RSV-associated acute lower respiratory infection in-hospital deaths in children aged 0–60 months. In infants aged 0–6 months, there were 6·6 million RSV-associated acute lower respiratory infection episodes, 1·4 million RSV-associated acute lower respiratoryinfection hospital admissions, and 13 300 RSV-associated acute lower respiratory infection in-hospital deaths. We highlighted the substantial unmeasured burden of RSV mortality, with one in every 50 deaths in children aged 0–60 months and one in every 28 deaths in children aged 28 days–6 months attributable to RSV. These findings suggest that RSV passive immunisation programmes targeting the first 6 months of life could have a substantial effect on reducing RSV morbidity and mortality burden.

Our estimates of RSV morbidity (including disease burden in the community and hospital) were broadly consistent with our previous estimates for the year 2015.^3^ The Global Burden of Diseases, Injuries, and Risk Factors Study (GBD) 2016^15^ reported 10·7 million acute lower respiratory infection episodes in children aged 0–60 months in all health-care settings. This was between our estimates of 3·6 million hospital admissions for RSV-associated acute lower respiratory infection (a narrower definition than that used in GBD 2016), and 33·0 million RSV-associated acute lower respiratory infection episodes (attending or not attending health care, a broader definition than that used in GBD 2016). The estimate of 3·6 million hospital admissions for RSV-associated acute lower respiratory infection in the present study is also broadly consistent with extrapolated global estimates (2·5–4·1 million) from the national estimates for 58 countries.^21^

In our previous estimates for 2015,^3^ we had to use imputed rates for children aged 0–6 months due to paucity of data. For example, our previous estimates of RSV-associated acute lower respiratory infection incidence in children aged 0–6 months for low-income and high-income countries were exclusively based on imputed rates from older age groups. Because infant age groups are crucial for RSV immunisation strategy, we made extensive efforts to identify and collect unpublished data on these age groups in this study. For children aged 0–6 months, we included 12 more community-based studies with RSV incidence data, more than double the number of studies in the previous study (22 *vs* ten); each of the income regions had available RSV data so imputation was not required in this study. Because of the expanded dataset, we could observe a larger gap in RSV disease burden between LMICs and high-income countries in children aged 0–6 months than in children aged 0–60 months. The disproportionately high RSV burden in the younger age groups in LMICs warrants more extensive community case management and effective and affordable immunisation programmes. The gap is even larger when it comes to hospital admission, reflecting the fact that access and availability to hospital care is still limited in LMICs. Despite higher incidence in the community, the RSV-associated acute lower respiratory infection hospital admission rate in LMICs was similar to that in high-income countries for children aged 0–60 months, and for children aged 0–6 months, astonishingly, the hospital admission rate in LMICs was consistently lower than that in high-income countries.

Since our previous estimates for 2015^3^, we have improved our methods to develop more robust estimates in our subsequent global disease burden studies for other viral infections.^9–11,22^ One important change is the use of GLMM framework in place of the conventional random-effects model (REM); compared with REM, GLMM has advantages when handling sparse data (ie, when case or denominator counts are small).^23^ This helps explain the differences in the RSV-associated acute lower respiratory infection in-hospital deaths between our present and previous estimates, as REM tends to bias CFR estimates upwards, towards 0·5,^23^ which inflated the CFR estimate for older children in particular. When applying the same model to our present and previous data, there was only moderate difference in the estimates. Nevertheless, the decrease in the in-hospital mortality estimates from previous estimates cannot be attributed entirely to the use of different models. Our stratified analysis (by the study median year, 2012) identified a decrease in in-hospital CFR over time, especially among LMICs, highlighting the overall improvement in quality of care in recent years. We observed some interesting trends for RSV-associated acute lower respiratory infection in-hospital CFR when stratified by age and income regions. Overall, in-hospital CFR of RSV-associated acute lower respiratory infection decreased with increase in income level. This could be due to several reasons. First, the paucity of appropriate care in the form of supportive management (eg, oxygen supplementation and suction of respiratory secretions) in LMICs. Second, differences in health-care accessibility and affordability in LMICs where children are likely to be more severely ill when brought to hospitals. In addition, CFR decreased with age in LMICs but did not change substantially over age in high-income countries; we were unable to observe such trends over age in our previous analysis,^3^ largely because of REM inflating the CFR estimates in older children. Results from our sensitivity analysis suggest that the gap in in-hospital CFR between LMICs and high-income countries has reduced but is still substantial.

We previously estimated that there were 118 200 RSV-associated deaths from acute lower respiratory infection in children aged 0–60 months in 2015, but this was based on limited data, through an indirect excess mortality approach that relied on the statistical correlation between RSV morbidity and acute lower respiratory infection mortality.^3^ In the present study, we developed new models that could incorporate all the RSV community mortality data that became available in the last 3 years. This has been a major advancement in understanding the previously unrecognised burden of overall RSV mortality in infants. For the first time, we were able to estimate RSV overall mortality burden by narrower age bands and by region. We decided to report RSV-attributable all-cause deaths as the primary estimate and RSV-associated all-cause and acute lower respiratory infection deaths as secondary estimates in this study, with several considerations. Compared with all-cause death, determination of acute lower respiratory infection as cause of death relied mostly on verbal autopsy; misclassification and recall bias related to verbal autopsy could affect the estimate,^24^ which was also reflected by our finding that RSV-associated acute lower respiratory infection deaths only accounted for 48% of RSV-associated all-cause deaths. Compared with RSV-associated death where RSV was identified in the upper respiratory tract, but not necessarily in the causal chain, using RSV-attributable death is more relevant for understanding the impact of RSV prophylactics on RSV mortality. As the primary measure, we estimated that there were RSV-attributable deaths accounted for 2% of all-cause deaths in children aged 0–60 months. Approximately 45% of these RSV-attributable deaths occurred in the first 6 months of life, with RSV being in the causal chain in about 4% of all-cause deaths for post-neonatal infants. Although not being a common cause of deaths in the neonatal period, RSV was in the causal chain for about 19% of RSV-attributable deaths in children aged 0–6 months. A study^25^ based on global case series of RSV mortality reported a greater proportion of RSV neonatal deaths in community than in hospitals. These findings highlight the need for RSV maternal vaccine or a birth-dose of RSV monoclonal antibody.

Based on the estimates for in-hospital and overall mortality above, we further showed that globally, only 26% of RSV-attributable deaths occurred in hospitals in children aged 0–60 months; that is three deaths in the community for every RSV-associated acute lower respiratory infection in-hospital death. This proportion is lower than the 50% that we estimated previously with limited data.^3^ The gap between in-hospital and community deaths is even more pronounced in low-income countries, with 19% of the RSV-attributable deaths occurring in hospitals (ie, four deaths in community for every in-hospital death). A study conducted in a remote rural area with poor access to care in India reported that for every RSV in-hospital death, there could be as many as 13 RSV community deaths.^26^ Most of the striking gap between in-hospital and community deaths in low-income settings can be explained by the poor access to care, cost of care, and limited beds in hospitals during an RSV epidemic.^27^ Another explanation is that some of the RSV deaths might be in children with rapidly progressive illness who, initially, do not appear to be severely ill. An RSV community mortality study in urban slums in Buenos Aires, Argentina, showed that home deaths could occur during sleep, with mild bronchiolitis or even without any apparent lung disease.^17^ This justifies our reporting of all-cause deaths over acute lower respiratory infection death for overall deaths estimation.

One of the major differences between in-hospital mortality data and community mortality data included in our study is the time of testing. RSV was often tested upon admission in hospital-based studies, whereas in community mortality studies, only post-mortem samples were tested. It is widely acknowledged that RSV could predispose individuals to secondary bacterial infection,^28–30^ the latter of which could be lethal. Therefore, it is likely that some of the RSV-attributable deaths could not be captured by post-mortem testing alone as RSV would probably be undetectable by the time of testing. An analysis of the Scottish health-care data^31^ suggests that deaths occurring up to 1 month following the initial RSV diagnosis could be attributable to RSV. This suggests that the estimated overall RSV mortality burden in our study could still be an underestimate of the true burden, which might only be quantified by vaccine probe studies. Moreover, some of the community mortality studies were done in under-resourced settings and therefore might not be fully representative of the country. This could affect our estimates if the proportion of RSV in all-cause and acute lower respiratory infection deaths differed by level of deprivation within that country.

Given the disproportionally high burden of RSV morbidity and mortality in children aged 0–6 months, passive immunisation programmes targeting the first 6 months of life could have a substantial effect on reducing RSV disease burden. For example, assuming that RSV passive immunisation could confer 70% protection to infants aged up to 5 months, then this could directly avert up to 864000 RSV-associated acute lower respiratory infection hospital admissions and 26 800 RSV-attributable deaths globally per year. Within the first 6 months of life, over 60% of the RSV-associated acute lower respiratory infection hospital admissions were during the first 3 months of life. This suggests that RSV immunisation products could still be impactful even with a shorter duration of protection, although they would probably miss the substantial RSV-associated acute lower respiratory infection incidence that only peaked during 3–6 months of age in low-income and lower-middle-income countries. Compared with children aged 0–6 months, the disease burden in children aged 6–12 months was smaller but still substantial, especially for morbidity burden. This suggests that further investment is warranted for RSV prophylactic products targeting this age group.

Our study had several limitations, as discussed previously.^3^ First, heterogeneities in factors such as study setting, exact case definition for acute lower respiratory infection, health-care access and seeking behaviour, eligibility for RSV testing and proportion of specimens tested, and RSV testing assay could affect our estimates, although sensitivity analyses based on the factors above that removed studies with high risks of bias showed broadly consistent estimates (appendix pp 44–48). Second, for both morbidity and mortality estimates, we were constrained by the data to break down the age bands any further or to model the year-on-year changes in the RSV disease burden; for which more data on multi-year changes in RSV morbidity and mortality are warranted. Third, we did not specifically report the burden of RSV-associated acute lower respiratory infection in primary care, such as general practice and outpatient. Fourth, we applied data imputation for estimating the RSV-associated acute lower respiratory infection incidence and hospital admission rates for children aged 0–60 months among studies that did not report data in specific age bands but reported data either overall for children aged 0–60 months or some of the narrower age bands; nonetheless, sensitivity analyses that excluded imputed rates did not yield substantial differences in the estimates. Fifth, the data for RSV overall mortality estimates were still scarce (15 studies) and mostly represented under-resourced settings. Finally, all of our included data were collected before the coronavirus disease 2019 (COVID-19) pandemic; reports from France,^32^ Iceland,^33^ and Australia^34^ showed that children hospitalised for RSV disease in the first wave of RSV epidemics following the onset of the COVID-19 pandemic were older than those in the pre-pandemic period. It is unknown how the COVID-19 pandemic could affect RSV disease burden in the long term. Our estimates could serve as a reference for understanding RSV epidemiology in the context of the ongoing COVID-19 pandemic.

Despite these limitations, our revised estimates are based on a substantially expanded dataset (including the community mortality data) and improved methodology. For the first time, we managed to break down the population of children aged 0–6 months into narrower age bands for both morbidity and mortality estimates that are essential for estimating the impact of RSV prophylactics. These estimates should provide a comprehensive global overview of RSV morbidity and mortality burden in infants and young children. With the numerous RSV prophylactic products in the pipeline, our estimates provide an important baseline profile of RSV disease burden for evaluating their potential clinical impact and cost-effectiveness of public health programmes.

## Supplementary Material

Supplementary appendix

## Figures and Tables

**Table 1:** Incidence and number of episodes of RSV-associated acute lower respiratory infection in children younger than 5 years in 2019, by World Bank income regions and development status

	Low income	Lower-middle income	Upper-middle income	High income	Developing countries	Industrialised countries	Global*
**RSV-associated acute lower respiratory infection**							
0–3 months							
Studies	2	7	5	3	14	3	17
Incidence rate	8·0 (4·8-13·6)	57·1 (22·6-144·4)	121·5 (55·9–264·1)	19·6 (6·5–597)	55–3 (27·1–113·0)	19·6 (6·5–59·7)	51·8 (277–105·6)
Number of episodes	49 000 (29000–82 000)	895000 (354000-2 263 000)	1085000 (499 000–2 357000)	66000 (22 000–200 000)	1699000 (832000–3469000)	65000 (21000-198000)	1763000 (941000–3592000)
3–6 months							
Studies	2	7	5	3	14	3	17
Incidence rate	82·9 (44·5-154·7)	142·2 (95·8–211·2)	91·6 (28·8–291·6)	17·9 (4·8–66·7)	116·1 (73·8–182·6)	17·9 (4·8–66·7)	106·4 (70·6–168·3)
Number of episodes	504000 (270000-939000)	2228000 (1501000–3 309 000)	817000 (257000–2 602 000)	60000 (16000–223000)	3564000 (2 266000–5607000)	59000 (16000–221000)	3620000 (2403 000–5726000)
0–6 months†							
Studies	5	7	6	4	18	4	22
Incidence rate	75·9 (42·7-134·7)	106·0 (63·5–177·0)	130·8 (56·8–300·8)	29·0 (12·9–65–0)	103·7 (70·0-153·6)	29·0 (12·9–65·0)	96·3 (67·9–142·6)
Number of episodes	921000 (518000–1636 000)	3323000 (1991000–5 547 000)	2334000 (1014000–5369000)	194000 (86 000–435 000)	6371000 (4302 000–9436000)	192000 (86 000–431000)	6554000 (4620000–9702000)
6–12 months							
Studies	5	8	6	4	19	4	23
Incidence rate	68·7 (31·5-15O·0)	105·4 (73·1–152·1)	84·3 (39·5–180·1)	32·5 (19·9–53·0)	88·2 (62·2–125·2)	32·5 (19·9–53·0)	82·6 (60·8–116·9)
Number of episodes	835000 (383000–1821000)	3303000 (2289000–4766000)	1505000 (705 000–3 215 000)	217000 (133000–354000)	5419000 (3820000–7689000)	215000 (132000–351000)	5 619000 (4135000–7953000)
0–12 months†							
Studies	5	8	6	5	19	5	24
Incidence rate	78·3 (43·2–142·2)	111·2 (81✓-151·2)	108·8 (48·6–243·7)	38·5 (21·6–68·8)	1010 (72·5-140·6)	38·5 (21·6–68·8)	94·6 (70·8–131·6)
Number of episodes	1902000 (1048 000-3453000)	6969000 (5123000–9480000)	3 885000 (1735000–8 698000)	515000 (288000–920000)	12 401000 (8 907000–17267000)	510000 (286000–911000)	12 875 000 (9635000–17909000)
12–60 months							
Studies	2	4	0	0	6	0	0
Incidence rate	35·9 (8·4–154·2)	25·4 (17·3–37·3)	..	..	27·7 (15·3–50·2)	.	.
Number of episodes	3307000 (770 000–14 208 000)	6274000 (4 273 000–9 212 000)	..	..	13500000 (7465 000–24413 000)	.	.
0–60 months†							
Studies‡	7(4)	9(4)	6(6)	7(5)	22(14)	7(5)	29(19)
Incidence rate	49·3 (29·4–82·8)	51·4 (37·8–69·8)	55·2 (25·4–119·9)	24·3 (13·8–427)	51·6 (38·1–69·9)	24·3 (13·8–42·7)	48·8 (37·4–65·9)
Number of episodes	5738000 (3418000–9633000)	15913 000 (11715 000–21616 000)	10079000 (4639000–21895000)	1657000 (943 000–2 914000)	31434000 (23194000–42601000)	1646 000 (936000–2 894000)	33028000 (25353000–44638000)
RSV-associated acute lower respiratory infection with chest wall indrawing							
0–3 months							
Studies	2	6	5	3	13	3	16
Incidence rate	2–0 (03–12·1)	45·6 (24·2–85·9)	75–6 (28·8–198·5)	4–0 (0·6–28·2)	30–9 (13·0–73·5)	4–0 (0·6–28·2)	28–3 (12·9–68·2)
Number of episodes	12 000 (2000–73 000)	714000 (379000–1345000)	675000 (257000–1771000)	13 000 (2000–94000)	949000 (399000–2256000)	13 000 (2000–93000)	963000 (439000–2321000)
3–6 months							
Studies	2	6	5	3	13	3	16
Incidence rate	18–1 (7·5–43·2)	40–6 (14·6–113·0)	14–1 (1·1–182·7)	4–0 (0·6–28·2)	27–6 (11·1–68·4)	4–0 (0·6–28·2)	25–3 (11·1–63·6)
Number of episodes	110000 (46 000–262 000)	636000 (229000–1770000)	126000 (10000–1630000)	13 000 (2000–94000)	848000 (342000–2101000)	13 000 (2000–93000)	862000 (379 000–2165 000)
0–6 months†							
Studies	4	6	5	3	15	3	18
Incidence rate	20–7 (6·8–62·9)	45·8 (23·7–88·4)	50–8 (15·5–166·2)	1–4 (1-3-1-5)	38–2 (21·1–69·1)	1–4 (1-3-1-5)	34–4 (19·9–62·7)
Number of episodes	251000 (82000–764000)	143 6 000 (744 000–2 769 000)	907000 (277000–2 966000)	9000 (9000–10000)	2348000 (1299000–4 247 000)	9000 (9000–10000)	2 343 000 (1352000–4267000)
6–12 months							
Studies	4	7	5	3	16	3	19
Incidence rate	13–1 (2·6–65·5)	27–8 (20·9–36·9)	9–7 (2·2–43·0)	12–6 (5·6–28·4)	17–4 (9·6–31·6)	12–6 (5·6–28·4)	16–9 (10·3–30·2)
Number of episodes	160000 (32000–796000)	871000 (656000-1i55000)	174 000 (39000–768000)	84000 (37000–190000)	1070000 (589000–1943000)	83000 (37000–188000)	1152 000 (698000–2 057000)
0–12 months†							
Studies	4	7	5	4	16	4	20
Incidence rate	19·5 (5·9–64·5)	36–7 (24·1–55·8)	31–0 (9·4–102·5)	9–0 (4·8–17·0)	30–0 (17·8–50·5)	9–0 (4·8–17·0)	27–9 (17·6–47·1)
Number of episodes	474000 (143000–1567000)	2300000 (1513 000–3 499 000)	1107000 (335000–3 657000)	121000 (64000–228000)	3 684000 (2187 000–6 206 000)	120000 (63000–225000)	3794000 (2394000–6405000)
12–60 months							
Studies	2	3	0	0	5	0	0
Incidence rate	1·4 (<0·05–38·5)	8–1 (3·0–22·2)			3–7 (0·7–19·0)		
Number of episodes	124000 (4000–3550000)	2007000 (733000–5495000)			1809000 (355000–9 227000)		
0–60 months†							
Studies‡	2	3	0	1	5	1	6
Incidence rate	4·8 (0·5–45·2)	14–0 (8·0–24·2)		3–1 (1·7–5-5)	8–8 (3·0–25·3)	3–1 (1·7–5-5)	8–1 (3·2–23·2)
Number of episodes	560000 (59 000–5 262 000)	4325000 (2492000–7507000)		208000 (116000–376000)	5341000 (1850 000–15 419 000)	207000 (115 000–374000)	5488000 (2192000–15744000)

Data are n, incidence rate per 1000 children per year (UR), or n (UR). RSV=respiratory syncytial virus. UR=uncertainty range. *Global estimates were obtained by summing the numbers of developing and industrialised countries for each of the 1000 samples in the Monte Carlo simulation. †The point estimates and uncertainty range estimates are not necessarily equal to the sum of the estimates by finer age bands; this is because the studies that contributed to different age-group-specific estimates were different. ‡Data in parentheses indicate the number of studies with imputed data; comparisons between estimates using imputed data and not using imputed data are presented in the appendix (p 44).

**Table 2:** Estimates of RSV-associated acute lower respiratory infection hospital admissions in children younger than 5 years by World Bank income regions and development status, 2019

	Low income	Lower-middle income	Upper-middle income	High income	Developing countries	Industrialised countries	Global*
**RSV-associated acute lower respiratory infection hospital admission**							
0–3 months							
Studies	6	11	16	19	36	16	52
Hospital admission rate	10·6 (3·3–33·5)	31·0 (17·0–56·4)	26·4 (12·8–54·5)	34·7 (21·5–56·2)	23·5 (15·2–36·3)	36·9 (20·9–65·0)	24·7 (17·5–37·1)
Number of episodes	64 000 (20 000–204 000)	485 000 (267 000–884 000)	236 000 (114 000–486 000)	116 000 (72 000–188 000)	721 000 (466 000–1 115 000)	122 000 (69 000–215 000)	841 000 (597 000–1 261 000)
3–6 months							
Studies	6	13	16	21	38	18	56
Hospital admission rate	6·0 (0·9–39·9)	19·2 (11·5–32·1)	20·6 (11·8–36·0)	20·7 (13·5–31·6)	16·7 (11·2–24·9)	20·6 (12·4–34·1)	17·0 (12·4–24·9)
Number of episodes	36 000 (5000–242 000)	301 000 (180 000–503 000)	184 000 (106 000–321 000)	69 000 (45 000–106 000)	513 000 (345 000–765 000)	68 000 (41 000–113 000)	579 000 (422 000–846 000)
0–6 months†							
Studies	10	12	16	27	41	24	65
Hospital admission rate	7·9 (2·8–21·9)	27·9 (16·7–46·6)	24·3 (13·2–44·7)	28·4 (20·2–40·0)	19·3 (13·1–28·6)	29·3 (20·0–42·8)	20·2 (14·9–29·1)
Number of episodes	96 000 (34 000–266 000)	873 000 (523 000–1 460 000)	434 000 (236 000–798 000)	190 000 (135 000–267 000)	1 188 000 (802 000–1 759 000)	194 000 (133 000–283 000)	1 376 000 (1 017 000–1 982 000)
6–12 months							
Studies	10	13	15	27	41	24	65
Hospital admission rate	5·7 (2·6–12·3)	12·1 (6·5–22·8)	12·1 (6·6–22·1)	11·2 (7·5–16·7)	10·0 (6·9–14·4)	11·1 (7·1–17·4)	10·0 (7·4–14·3)
Number of episodes	69 000 (32 000–150 000)	381 000 (203 000–715 000)	215 000 (117 000–394 000)	75 000 (50 000–112 000)	612 000 (422 000–886 000)	74 000 (47 000–116 000)	683 000 (507 000–973 000)
0–12 months†							
Studies	13	20	15	41	51	38	89
Incidence rate	9·6 (5·0–18·7)	17·5 (11·5–26·5)	18·7 (10·2–34·5)	22·0 (17·1–28·4)	15·3 (11·3–20·8)	22·5 (17·1–29·5)	15·9 (12·6–21·2)
Number of episodes	234 000 (120 000–455 000)	1 095 000 (722 000–1 661 000)	669 000 (363 000–1 232 000)	294 000 (228 000–380 000)	1 881 000 (1 386 000–2 552 000)	298 000 (227 000–391 000)	2 170 000 (1 713 000–2 882 000)
12–60 months							
Studies	9	12	8	17	31	15	46
Hospital admission rate	1·6 (0·5–4·6)	1·6 (1·0–2·7)	1·5 (0·8–2·8)	1·6 (1·2–2·1)	1·5 (1·0–2·3)	1·7 (1·3–2·3)	1·5 (1·1–2·2)
Number of episodes	145 000 (50 000–421 000)	396 000 (235 000–667 000)	220 000 (117 000–415 000)	88 000 (67 000–116 000)	735 000 (491 000–1 101 000)	95 000 (72 000–125 000)	827 000 (600 000–1 207 000)
0–60 months†							
Studies‡	16 (4)	22 (6)	16 (8)	51 (28)	57 (19)	48 (27)	105 (46)
Hospital admission rate	3·5 (2·0–6·3)	6·2 (4·0–9·4)	6·2 (3·8–10·3)	6·0 (4·7–7·7)	5·2 (3·9–6·9)	6·1 (4·7–7·9)	5·3 (4·2–6·8)
Number of episodes	411 000 (231 000–731 000)	1 908 000 (1 251 000–2 909 000)	1 139 000 (693 000–1 872 000)	409 000 (319 000–524 000)	3 163 000 (2 395 000–4 179 000)	413 000 (318 000–537 000)	3 567 000 (2 856 000–4 634 000)
**RSV-associated acute lower respiratory infection hospital admission with hypoxaemia**							
0–3 months							
Studies	9	7	15	9	32	8	40
Hospital admission rate	2·5 (0·4–14·9)	5·7 (1·5–18·0)	9·2 (2·4–29·9)	12·4 (2·7–40·7)	6·9 (3·1–15·4)	9·4 (1·4–41·3)	7·4 (3·6–15·5)
Number of episodes	15 000 (2000–90 000)	90 000 (24 000–282 000)	82 000 (21 000–267 000)	42 000 (9000–136 000)	211 000 (95 000–473 000)	31 000 (5000–137 000)	252 000 (121 000–527 000)
3–6 months							
Studies	9	10	15	10	35	9	44
Hospital admission rate	1·0 (0·1–12·4)	3·2 (1·1–7·9)	6·3 (2·0–17·4)	7·9 (1·9–22·9)	4·0 (1·9–8·6)	5·5 (1·0–21·3)	4·2 (2·1–8·6)
Number of episodes	6000 (0–76 000)	50 000 (18 000–123 000)	56 000 (17 000–155 000)	26 000 (6000–77 000)	122 000 (57 000–263 000)	18 000 (3000–70 000)	144 000 (73 000–293 000)
0–6 months†							
Studies	9	7	15	10	32	9	41
Hospital admission rate	1·5 (0·3–7·8)	4·5 (1·5–11·9)	8·3 (2·5–23·7)	10·9 (3·0–28·6)	5·2 (2·5–11·2)	8·2 (1·7–26·6)	5·7 (2·9–11·3)
Number of episodes	19 000 (3000–95 000)	142 000 (47 000–372 000)	149 000 (44 000–423 000)	73 000 (20 000–191 000)	322 000 (153 000–688 000)	54 000 (11 000–176 000)	385 000 (198 000–768 000)
6–12 months							
Studies	9	10	15	10	35	9	44
Hospital admission rate	0·9 (0·2–3·7)	2·0 (0·7–5·3)	3·0 (0·6–10·8)	5·0 (1·4–12·8)	2·2 (1·0–4·9)	3·5 (0·8–11·1)	2·3 (1·2–4·9)
Number of episodes	11 000 (2000–45 000)	62 000 (21 000–167 000)	53 000 (11 000–193 000)	33 000 (10 000–85 000)	133 000 (59 000–301 000)	23 000 (5000–74 000)	159 000 (79 000–336 000)
0–12 months†							
Studies	9	11	15	10	36	9	45
Hospital admision rate	1·7 (0·4–6·2)	3·9 (1·6–8·7)	6·0 (1·7–17·9)	8·9 (2·8–20·8)	4·1 (2·2–7·9)	6·6 (1·7–18·5)	4·5 (2·5–8·0)
Number of episodes	42 000 (11 000–151 000)	245 000 (98 000–543 000)	214 000 (60 000–639 000)	120 000 (37 000–279 000)	509 000 (268 000–976 000)	88 000 (22 000–245 000)	606 000 (342 000–1 095 000)
12–60 months							
Studies	8	8	9	8	26	7	33
Hospital admission rate	0·1 (<0·05–1·0)	0·2 (<0·05–0·8)	0·3 (<0·05–1·9)	0·6 (0·1–1·6)	0·2 (0·1–0·7)	0·4 (0·1–1·4)	0·2 (0·1–0·7)
Number of episodes	13 000 (2000–92 000)	52 000 (10 000–209 000)	50 000 (5000–272 000)	34 000 (8000–89 000)	106 000 (32 000–346 000)	24 000 (5000–76 000)	134 000 (51 000–383 000)
0–60 months†							
Studies‡	8	8	9	8	26	7	33
Hospital admission rate	0·5 (0·1–1·7)	1·3 (0·4–3·3)	2·1 (0·4–7·2)	2·3 (0·6–5·9)	1·3 (0·6–2·8)	1·5 (0·3–4·8)	1·3 (0·7–2·8)
Number of episodes	56 000 (15 000–198 000)	406 000 (138 000–1 009 000)	385 000 (67 000–1 313 000)	159000 (41 000–401 000)	795 000 (361 000–1 710 000)	102 000 (21 000–328 000)	911 000 (459 000–1 866 000)

Data are n, hospital admission rate per 1000 children per year (UR), or n (UR). Number of episodes were were rounded to the nearest 1000. RSV=respiratory syncytial virus. UR=uncertainty range. *Global estimates were obtained by summing the numbers of developing and industrialised countries for each of the 1000 samples in the Monte Carlo simulation. †The point estimates and uncertainty range estimates are not necessarily equal to the sum of the estimates by finer age bands; this is because the studies that contributed to different age-group-specific estimates were different. ‡Data in parentheses indicate the number of studies with imputed data; comparisons between estimates using imputed data and not using imputed data are presented in the appendix (p 44).

**Table 3:** CFR estimates and number of in-hospital deaths in children younger than 5 years with RSV-associated acute lower respiratory infection in 2019, by World Bank income regions and development status

	Low income	Lower-middle income	Upper-middle income	High income	Developing countries	Industrialised countries	Global*
**0–3 months**							
Studies	15	15	20	18	52	16	68
In-hospital CFR (%)	2·6 (1·8–3·6)	1·8 (0·8–3·6)	0·7 (0·4–1·4)	<0·05 (<0·05–0·3)	1·1 (0·7–1·8)	<0·05 (<0·05–0·2)	1·0 (0·6–1·6)
Number of deaths	1600 (300–7800)	8500 (2100–31 300)	1800 (400–6700)	<50 (0–500)	8000 (3500–19 800)	<50 (0–500)	8100 (3600–20 100)
**3–6 months**							
Studies	15	20	20	17	57	15	72
In-hospital CFR (%)	2·2 (1·5–3·3)	1·0 (0·4–2·5)	0·7 (0·3–1·7)	<0·05 (<0·05–0·1)	0·9 (0·6–1·6)	<0·05 (<0·05–0·1)	0·8 (0·5–1·4)
Number of deaths	800 (100–8900)	3000 (600–12 200)	1300 (300–5500)	<50 (0–100)	4700 (2000–12 000)	<50 (0–200)	4800 (2100–12 100)
**0–6 months†**							
Studies	16	17	21	17	56	15	71
In-hospital CFR (%)	2·4 (1·8–3·1)	1·7 (1·0–2·8)	0·8 (0·4–1·3)	<0·05 (<0·05–0·2)	1·1 (0·8–1·6)	<0·05 (<0·05–0·2)	1·0 (0·7–1·4)
Number of deaths	2300 (600–8800)	15 100 (5200–40 500)	3400 (1100–10 300)	100 (0–500)	13 200 (6600–27 800)	<50 (0–500)	13 300 (6800–28 100)
**6–12 months**							
Studies	17	20	21	17	60	15	75
In-hospital CFR (%)	1·8 (0·9–3·4)	0·8 (0·3–2·1)	0·4 (0·2–1·2)	0·1 (<0·05–0·3)	0·8 (0·5–1·3)	<0·05 (<0·05–0·2)	0·7 (0·4–1·2)
Number of deaths	1200 (300–5400)	3100 (600–14 500)	1000 (200–4800)	<50 (0–400)	4800 (2100–11 600)	<50 (0–200)	4900 (2200–11 700)
**0–12 months†**							
Studies	18	22	27	29	70	26	96
In-hospital CFR (%)	1·5 (0·8–2·8)	1·5 (0·7–3·2)	0·8 (0·5–1·3)	0·1 (0·1–0·3)	1·1 (0·8–1·5)	0·1 (0·1–0·3)	0·9 (0·7–1·4)
Number of deaths	3500 (900–13 100)	16 500 (4700–52 100)	5600 (2000–15 600)	400 (200–1100)	20 100 (10 900–39 100)	300 (100–1000)	20 500 (11 300–39 800)
**12–60 months**							
Studies	16	18	20	17	58	13	71
In-hospital CFR (%)	1·6 (0·4–5·7)	0·8 (0·3–1·9)	0·1 (<0·05–0·8)	0·2 (0·1–0·4)	0·5 (0·3–1·0)	0·1 (0·1–0·1)	0·5 (0·3–0·9)
Number of deaths	2300 (200–25 400)	3200 (700–12 300)	300 (0–3200)	200 (100–500)	3800 (1 500–10 900)	100 (100–200)	3900 (1600–11 100)
**0–60 months†**							
Studies	19	26	30	26	77	24	101
In-hospital CFR (%)	1·4 (0·6–2·8)	0·8 (0·4–1·5)	0·6 (0·3–1·0)	0·1 (0·1–0·2)	0·8 (0·6–1·2)	0·1 (0·1–0·2)	0·7 (0·5–1·1)
Number of deaths	5600 (1400–21 300)	14 600 (4400–44 100)	6800 (2400–19 000)	500 (300–1300)	25 900 (14 500–48 600)	400 (200–900)	26 300 (15 100–49 100)

Data are n, CFR (UR), or n (UR). Numbers of deaths were rounded to the nearest hundred, except when the estimate was <50. RSV=respiratory syncytial virus. CFR=case fatality ratio. UR=uncertainty range. *Global estimates were obtained by summing the numbers of developing and industrialised countries for each of the 1000 samples in the Monte Carlo simulation. †The point estimates and uncertainty range estimates are not necessarily equal to the sum of the estimates by finer age bands; this is because the studies that contributed to different age-group-specific estimates were different.

**Table 4:** Estimates (with 95% uncertainty range) of RSV-attributable deaths, and RSV-associated all-cause and acute lower respiratory infection deaths in children younger than 5 years in 2019, by World Bank income regions and development status

	Low income	Lower-middle income	Upper-middle income	High income	Developing countries	Industrialised countries	Global*
**RSV-attributable† deaths (primary measure)**							
0–27 days							
Proportion (%), in all-cause deaths	0·8 (0·6–0·9)	0·7 (0·6–1·0)	0·6 (0·5–0·9)	0·6 (0·4–0·9)	0·7 (0·6–0·9)	0·6 (0·4–1·0)	0·7 (0·6–0·9)
Number of deaths	2600 (2100–3200)	4700 (3500–6400)	1000 (800–1500)	200 (200–400)	8300 (6900–10 100)	200 (200–400)	8500 (7100–10 400)
28 days to 6 months							
Proportion (%), in all-cause deaths	3·8 (3·2–4·6)	3·6 (2·7–4·9)	3·2 (2·5–4·2)	2·9 (2·2–4·2)	3·6 (3·0–4·4)	2·8 (2·1–4·3)	3·6 (3·0–4·4)
Number of deaths	10 000 (8400–12 200)	22 300 (16 900–30 300)	4100 (3200–5500)	500 (400–800)	36 700 (30 800–45 100)	500 (300–700)	37 200 (31 200–45 500)
0–6 months							
Proportion (%), in all-cause deaths	2·1 (1·8–2·6)	2·2 (1·6–2·9)	1·8 (1·4–2·4)	1·3 (1·0–2·0)	2·1 (1·8–2·6)	1·2 (0·9–2·0)	2·1 (1·7–2·5)
Number of deaths	12 600 (10 500–15 300)	27 000 (20 400–36 300)	5100 (4000–6900)	700 (500–1100)	45 000 (37 800–55 300)	700 (500–1100)	45 700 (38 400–55 900)
6–12 months							
Proportion (%), in all-cause deaths	2·6 (2·1–3·2)	2·4 (1·8–3·5)	2·1 (1·6–2·9)	2·0 (1·4–2·8)	2·5 (2·0–3·1)	1·9 (1·4–2·9)	2·5 (2·0–3·1)
Number of deaths	6300 (5100–8000)	12 500 (9100–17 800)	1600 (1200–2100)	100 (100–200)	20 500 (16 700–25 900)	100 (100–100)	20 600 (16 800–26 000)
0–12 months							
Proportion (%), in all-cause deaths	2·2 (1·9–2·8)	2·2 (1·7–3·1)	1·8 (1·4–2·5)	1·4 (1·0–2·0)	2·2 (1·8–2·7)	1·3 (0·9–2·0)	2·2 (1·8–2·7)
Number of deaths	18 900 (15 700–23 300)	39 600 (29 400–54 100)	6700 (5200–9000)	900 (600–1300)	65 500 (54 600–81 200)	800 (600–1200)	66 300 (55 200–82 000)
12–60 months							
Proportion (%), in all-cause deaths	1·7 (1·4–2·1)	1·6 (1·2–2·2)	1·4 (1·1–1·9)	1·3 (1·0–1·8)	1·6 (1·4–2·0)	1·2 (0·9–1·9)	1·6 (1·4–2·0)
Number of deaths	11 200 (9200–13 900)	21 300 (15 900–29 300)	2400 (1800–3200)	200 (100–200)	35 000 (29 000–43 600)	100 (100–200)	35 100 (29 100–43 600)
0–60 months							
Proportion (%), in all-cause deaths	2·0 (1·6–2·5)	2·0 (1·5–2·7)	1·7 (1·3–2·3)	1·4 (1·0–2·0)	2·0 (1·6–2·4)	1·3 (0·9–2·0)	2·0 (1·6–2·4)
Number of deaths	30 100 (24 900–37 200)	60 900 (45 300–84 300)	9100 (7000–12 100)	1000 (700–1500)	100 500 (83 600–124 500)	900 (600–1400)	101 400 (84 500–125 200)
**RSV-associated all-cause deaths (secondary measure)**							
0–27 days							
Proportion (%), in all-cause deaths	1·8 (1·5–2·1)	1·7 (1·3–2·2)	1·4 (1·1–1·9)	1·3 (1·0–1·9)	1·7 (1·5–2·0)	1·3 (1·0–1·9)	1·7 (1·5–2·0)
Number of deaths	5900 (5100–7000)	10 800 (8500–14 000)	2300 (1800–3100)	500 (400–700)	19 100 (16 400–22 300)	500 (400–800)	19 600 (16 900–22 900)
28 days to 6 months							
Proportion (%), in all-cause deaths	8·4 (7·3–9·8)	8·1 (6·4–10·1)	7·0 (5·8–8·6)	6·4 (5·1–8·4)	8·0 (7·0–9·4)	6·3 (4·9–8·6)	8·0 (7·0–9·3)
Number of deaths	22 200 (19 300–25 800)	49 800 (39 800–62 300)	9200 (7600–11 300)	1200 (900–1500)	81 400 (70 800–95 300)	1000 (800–1400)	82 500 (71 700–96 300)
0–6 months							
Proportion (%), in all-cause deaths	4·7 (4·1–5·5)	4·8 (3·9–6·1)	3·9 (3·3–4·9)	3·0 (2·3–4·0)	4·7 (4·1–5·5)	2·8 (2·1–3·9)	4·6 (4·0–5·4)
Number of deaths	28 100 (24 400–32 800)	60 700 (48 300–76 500)	11 400 (9500–14 300)	1700 (1300–2200)	100 500 (87 400–117 400)	1600 (1200–2200)	102 000 (88 800–118 800)
6–12 months							
Proportion (%), in all-cause deaths	5·7 (4·8–7·0)	5·5 (4·2–7·4)	4·8 (3·8–6·2)	4·5 (3·5–5·9)	5·5 (4·6–6·7)	4·2 (3·2–6·0)	5·5 (4·6–6·7)
Number of deaths	14 300 (12 000–17 300)	28 300 (21 500–37 800)	3500 (2800–4500)	300 (200–300)	46 200 (38 800–56 200)	200 (100–200)	46 400 (38 900–56 300)
0–12 months							
Proportion (%), in all-cause deaths	5·0 (4·3–5·9)	5·0 (4·0–6·5)	4·1 (3·4–5·1)	3·1 (2·4–4·1)	4·9 (4·2–5·8)	2·9 (2·2–4·1)	4·9 (4·2–5·8)
Number of deaths	42 300 (36 300–50 100)	89 000 (69 900–114 600)	15 000 (12 300–18 700)	1900 (1500–2500)	146 600 (126 200–173 400)	1700 (1300–2400)	148 500 (128 000–174 900)
12–60 months							
Proportion (%), in all-cause deaths	3·9 (3·3–4·7)	3·7 (2·9–4·9)	3·2 (2·6–4·1)	3·0 (2·3–3·9)	3·7 (3·2–4·5)	2·8 (2·2–3·9)	3·7 (3·2–4·5)
Number of deaths	25 600 (21 700–30 800)	48 800 (37 800–64 600)	5400 (4400–6800)	400 (300–500)	80 300 (67 700–96 400)	200 (200–300)	80 600 (68 000–96 600)
0–60 months							
Proportion (%), in all-cause deaths	4·5 (3·8–5·4)	4·5 (3·5–5·8)	3·8 (3·1–4·8)	3·1 (2·4–4·1)	4·4 (3·8–5·3)	2·9 (2·2–4·1)	4·4 (3·8–5·2)
Number of deaths	67 900 (58 000–80 900)	137 800 (107 600–178 800)	20 500 (16 700–25 500)	2300 (1800–3000)	226 800 (194 000–269 400)	2000 (1500–2800)	229000 (196 000–271 200)
**RSV-associated acute lower respiratory infection deaths (secondary measure)**							
0–27 days							
Proportion	7·4 (6·4–8·6)	7·4 (6·0–9·5)	8·3 (6·5–11·3)	8·8 (7·3–10·8)	7·4 (6·6–8·6)	8·9 (7·2–11·6)	7·4 (6·6–8·6)
Number of deaths	1500 (1300–1800)	2000 (1600–2600)	200 (200–300)	<50 (<50-<50)	3800 (3300–4400)	<50 (<50-<50)	3800 (3400–4400)
28 days to 6 months							
Proportion	16·6 (14·7–18·6)	17·1 (14·4–20·7)	18·1 (15·5–21·2)	18·4 (15·0–22·9)	17·0 (15·2–19·4)	19·5 (16·2–23·4)	17·1 (15·2–19·4)
Number of deaths	15 100 (13 500–17 000)	36 100 (30 300–43 700)	6000 (5100–7000)	300 (300–400)	57 300 (51 100–65 300)	200 (100–200)	57 500 (51 200–65 400)
0–6 months							
Proportion	14·9 (13·2–16·7)	16·0 (13·5–19·4)	17·3 (14·8–20·3)	17·6 (14·3–21·8)	15·8 (14·1–17·9)	17·7 (14·6–21·3)	15·8 (14·1–17·9)
Number of deaths	16 700 (14 800–18 700)	38 000 (32 000–46 100)	6200 (5300–7300)	300 (300–400)	61 100 (54 500–69 200)	200 (200–200)	61 300 (54 700–69 400)
6–12 months							
Proportion	10·5 (9·0–12·2)	10·8 (8·7–13·5)	11·6 (9·6–14·2)	12·0 (9·6–15·7)	10·7 (9·4–12·5)	12·5 (10·0–16·2)	10·7 (9·4–12·5)
Number of deaths	9000 (7800–10 500)	17 200 (14 000–21 600)	2300 (1900–2800)	100 (100–200)	28 500 (25 000–33 100)	100 (100–100)	28 700 (25 100–33 200)
0–12 months							
Proportion	13·0 (11·4–14·7)	13·9 (11·6–17·0)	15·3 (12·9–18·2)	15·4 (12·5–19·2)	13·7 (12·2–15·7)	15·4 (12·6–19·1)	13·7 (12·2–15·6)
Number of deaths	25 700 (22 500–29 200)	55 300 (46 000–67 500)	8500 (7200–10 200)	500 (400–600)	89 700 (79 600–102 200)	300 (200–400)	89 900 (79 800–102 400)
12–60 months							
Proportion	7·3 (6·3–8·5)	7·5 (6·2–9·2)	8·2 (6·7–10·3)	8·7 (7·2–10·8)	7·5 (6·6–8·6)	8·8 (7·0–11·8)	7·5 (6·6–8·6)
Number of deaths	6300 (5400–7200)	11 200 (9200–13 900)	2000 (1700–2500)	200 (200–200)	19 500 (17 200–22 300)	200 (200–300)	19 700 (17 400–22 500)
0–60 months							
Proportion	11·3 (9·9–12·9)	12·2 (10·1–14·9)	13·1 (11·0–15·7)	12·6 (10·3–15·5)	12·0 (10·6–13·6)	11·7 (9·5–15·0)	12·0 (10·6–13·6)
Number of deaths	32 000 (27 900–36 500)	66 600 (55 300–81 600)	10 500 (8900–12 700)	700 (600–800)	109 100 (96 700–124 400)	500 (400–600)	109 600 (97 200–124 900)

Data are n, proportion (%) in acute lower respiratory infection deaths (UR), or n (UR). Number of deaths were rounded to the nearest 100, except when the estimate was <50. RSV=respiratory syncytial virus. UR=uncertainty range. *Global estimates were obtained by summing the numbers of developing and industrialised countries for each of the 1000 samples in the Monte Carlo simulation. †RSV-attributable death is defined as RSV being anywhere in the causal chain of death.^5^

## Data Availability

The study data and R codes for the analysis are freely available in Edinburgh DataShare (https://doi.org/10.7488/ds/3138).

## Research in context

### Evidence before this study

Respiratory syncytial virus (RSV) is the main cause of acute lower respiratory infection among young children. We searched PubMed for studies published between Jan 1, 1995, and Oct 31, 2021, that reported global estimates of RSV morbidity and mortality in young children, using the search terms “(“Respiratory syncytial virus*” OR “RSV”) AND (“incidence*” OR “mortality” OR “death*” OR “morbidity” OR “burden”) AND (“child*” OR “pediatric” OR “paediatric” OR “infant*”)”. We previously estimated that in 2015, there were 33·1 million acute lower respiratory infection episodes, 3·2 million hospital admissions for acute lower respiratory infection and 59 600 acute lower respiratory infection in-hospital deaths associated with RSV in children aged 0–60 months; we also reported an estimated 118 200 overall deaths (ie, in-hospital and out-of-hospital or community deaths), derived indirectly based on in-hospital deaths. The Global Burden of Disease study by the Institute of Health Metrics and Evaluation estimated that 10·7 million acute lower respiratory infection episodes and over 41 000 acute lower respiratory infection deaths in children aged 0–60 months in 2016 were attributable to RSV. In a separate study, we estimated the national burden of RSV hospitalisation for 58 countries and reported an extrapolated estimate of 2·5–4·1 million RSV-associated hospitalisations globally in children aged 0–60 months in 2019. No robust estimates of RSV overall deaths were available from existing global studies that incorporated RSV community mortality data.

### Added value of this study

We substantially expanded our global RSV burden dataset by including 113 new studies from the literature review update and new unpublished data from 51 studies shared through RSV GEN. With these data and improved methods, we have now been able to estimate RSV morbidity and mortality burden for hospital settings and overall, and focus on narrower infant age groups that are targeted by RSV prophylactics in development. With data on RSV community mortality, we estimated the overall RSV mortality burden in different age groups–one death was attributable to RSV in every 50 deaths in children aged 0–60 months and in every 28 deaths in children aged 28 days to 6 months. For every RSV-associated acute lower respiratory infection in-hospital death, there were approximately three deaths attributable to RSV in the community.

### Implications of all the available evidence

By incorporating the latest RSV community mortality data, we provide robust estimates of global and regional burden of RSV-related mortality. Our findings, together with other existing RSV disease burden estimates, provide a comprehensive global overview of RSV morbidity and mortality burden in infants and young children. With many RSV prophylactic products in the pipeline, our estimates by narrower age bands provide important baseline information to the introduction, prioritisation, and evaluation of these products. Further evidence is warranted to understand the implications of the potential age-shifts in peak RSV burden to older age after administration of RSV prophylactics to infants younger than 6 months.
