# Supplementary material for: Global, regional, and national disease burden estimates of acute lower respiratory infections due to respiratory syncytial virus in children younger than 5 years in 2019: a systematic analysis
Source: Lancet. Author manuscript; Available in PMC 2022 Sep 8. (PMC7613574; doi:10.1016/S0140-6736(22)00478-0)
Supplement: Supplementary appendix [file EMS153575-supplement-Supplementary_appendix.pdf]

Supplementary Material

Search Strategy

Antimicrobial Therapy of Neonates with Necrotizing Enterocolitis: A Systematic Review

Four databases: MEDLINE (Ovid MEDLINE(R) ALL form 1946 to May 31, 2020) EMBASE (Embase 1974 to May 31, 2020), Cochrane CENTRAL (Issue 12 of 12, May 31, 2020) and CINHAL (CINHAL 1982 to May 31, 2020).

Search was run on May 31, 2020.

MEDLINE

#1 exp Enterocolitis, Necrotizing/ or nec.mp.
#2 anti?bioti*.mp.
#3 antibiotic.mp. or exp Anti-Bacterial Agents/
#4 exp Anti-Bacterial Agents/ or exp Anti-Infective Agents/ or anti infective.mp.
#5 antimicrobial.mp.
#6 anti microbial.mp.
#7 anti?biot* or anti?infect* or anti?bact* or anti?microb*
#8 2 or 3 or 4 or 5 or 6 or 7
#9 neonate.mp. or exp Infant, Newborn/
#10 infant.mp. or exp Infant/
#11 newborn.mp.
#12 9 or 10 or 11
#13 1 and 8 and 12

EMBASE

#1 exp Enterocolitis, Necrotizing/ or nec.mp.
#2 anti?bioti*.mp.
#3 antibiotic.mp. or exp Anti-Bacterial Agents/
#4 exp Anti-Bacterial Agents/ or exp Anti-Infective Agents/ or anti infective.mp.
#5 antimicrobial.mp.
#6 anti microbial.mp.
#7 anti?biot* or anti?infect* or anti?bact* or anti?microb*
#8 2 or 3 or 4 or 5 or 6 or 7
#9 neonate.mp. or exp Infant, Newborn/
#10 infant.mp. or exp Infant/
#11 newborn.mp.
#12 9 or 10 or 11
#13 1 and 8 and 12

Cochrane Central

#1 necrotizing enterocolitis:ti,ab,kw
#2 nec:ti,ab,kw
#3 MeSH descriptor: [Enterocolitis, Necrotizing] explode all trees
#4 #1 or #2 or #3
#5 antibiotic:ti,ab,kw
#6 Anti-Bacterial Agents:ti,ab,kw
#7 Anti-Infective Agents:ti,ab,kw
#8 anti infective:ti,ab,kw
#9 antimicrobial:ti,ab,kw
#10 anti microbial:ti,ab,kw
#11 MeSH descriptor: [Anti-Bacterial Agents] explode all trees
#12 MeSH descriptor: [Anti-Infective Agents] explode all trees
#13 #5 or #6 or #7 or #8 or #9 or 10 or #11 or #12
#14 neonate:ti,ab,kw
#15 infant:ti,ab,kw
#16 newborn:ti,ab,kw

© 2021. Thieme. All rights reserved.

#17 MeSH descriptor: [Infant, Newborn] explode all trees
#18 #14 or #15 or #16 or #17
#19 #4 and #13 and #18

CINHAL

#S14 S3 AND S9 ANS S13
#S13 S10 OR S11 OR S12
#S12 “newborn”
#S11 “infant”
#S10 “neonate”
#S9 S4 OR S5 OR S6 OR S7 OR S8
#S8 “antimicrobial”
#S7 “anti microbial”
#S6 “anti-infective agents”
#S5 “anti-bacterial agents”
#S4 “antibiotic”
#S3 S1 OR S2
#S2 “nec”
#S1 (MH “Enterocolitis, Necrotizing”)

Risk of bias: Faix et al,[18] a randomized, control trial of parenteral clindamycin in neonatal necrotizing enterocolitis

Bias domain	Source of bias	Support for judgment	Review authors’ judgment (assess as low, unclear or high risk of bias)
Selection bias	Random sequence generation	Randomization of treatment assignment was made by sampling without replacement	Low
	Allocation concealment	PZ randomized from sealed envelopes<br>PZ randomly assigned to the control A + G group could have c added to the treatment<br>5 patients had bacteremia at the time of diagnosis	High
Performance bias	Blinding of participants and personnel	Personnel was not blind (open label)<br>All clinical decision were made by physicians other than the investigators	Unclear
Detection bias	Blinding of outcome assessment	Abdominal radiograph were interpreted by attending pediatric radiologists who were unaware of the treatment assignment	Low
Attrition bias	Incomplete outcome data	Outcome data are complete<br>Two patient received additional vancomycin treatment (not excluded from the analysis)<br>PZ randomly assigned to the control A + G group could have c added to the treatment > included in the control group and not excluded	Unclear
Reporting bias	Selective reporting	Abdominal radiograph were interpreted by attending pediatric radiologists who were unaware of the treatment assignment<br>all clinical decision were made by physicians other than the investigators > more than one	Unclear
Other bias	Anything else, ideally prespecified	oral feeding<br>Apgar’s score at 5 min<br>Prenatal exposure to dexamethasone<br>Concomitant medication<br>Race<br>Concomitant pathologies<br>Bacterial resistance	High

 © 2021. Thieme. All rights reserved.

Risk of bias: Hansen et al,[14] a randomized, control study of oral gentamicin in the treatment of neonatal necrotizing enterocolitis

Bias domain	Source of bias	Support for judgment	Review authors' judgment (assess as low, unclear or high risk of bias)
Selection bias	Random sequence generation	Patient assigned randomly	Low
	Allocation concealment	Patient assigned by a random number table	Low
Performance bias	Blinding of participants and personnel	The control group received placebo instead of gentamicin > not written if it was blind neither double-blind	Unclear
Detection bias	Blinding of outcome assessment	Not described	Unclear
Attrition bias	Incomplete outcome data	One patient with sepsis and meningitis who received just a single dose and died was excluded -> not clear from the analysis (excluded just in some analysis)	Unclear
Reporting bias	Selective reporting	Patients clinical status was assessed frequently by one of the authors<br>No clearly stated outcomes	High
Other bias	Anything else, ideally prespecified	Small sample size<br>oral feeding<br>Prenatal exposure to dexamethasone<br>Concomitant medication<br>Race<br>Concomitant pathologies<br>Bacterial resistance	High

ROBINS-I tool—comparison of two antibiotic regimens for neonatal necrotizing enterocolitis

ROBINS-I tool (stage II): for each study—comparison of two antibiotic regimens for neonatal necrotizing enterocolitis (Scheifele et al[19])

Specify a target randomized trial specific to the study

Design	Individually randomized/cluster randomized/matched (e.g., crossover) nonrandomized
Participants	90 infants
Experimental intervention	Cefotaxime + vancomycin
Comparator	Ampicillin + gentamicin

Is your aim for this study …?

✓	To assess the effect of *assignment to* intervention
☐	To assess the effect of *starting and adhering to* intervention

 © 2021. Thieme. All rights reserved.

Specify the outcome

Specify which outcome is being assessed for risk of bias (typically from among those earmarked for the Summary of Findings table). Specify whether this is a proposed benefit or harm of intervention.

Case population definition (inclusion exclusion criteria), aim, collection of data, study design, and statistical analysis

Specify the numerical result being assessed

In case of multiple alternative analyses being presented, specify the numeric result (e.g., RR = 1.52 [95% CI 0.83–2.77]) and/or a reference (e.g., to a table, figure, or paragraph) that uniquely defines the result being assessed.

Preliminary consideration of confounders

Complete a row for each important confounding domain (1) listed in the review protocol; and (2) relevant to the setting of this particular study, or which the study authors identified as potentially important.

"Important" confounding domains are those for which, in the context of this study, adjustment is expected to lead to a clinically important change in the estimated effect of the intervention

(1) Confounding domains listed in the review protocol				
**Confounding domain**	**Measured variable(s)**	**Is there evidence that controlling for this variable was unnecessary?**	**Is the confounding domain measured validly and reliably by this variable (or these variables)?**	**Optional: is failure to adjust for this variable (alone) expected to favor the experimental intervention or the comparator?**
			Yes/no/no information	Favor experimental/favor comparator/no information
Concomitant pathology				
Concomitant spontaneous intestinal perforation				
Concomitant medication				
Concomitant infections				
Birth weight				
Assisted ventilation				
Oral feeding				
Prenatal exposure to dexamethasone				
Gestational age				
Apgar's score at 5 min				
Highest level of fraction of inspired oxygen				
Inotropic support				

Note: "Validity" refers to whether the confounding variable or variables fully measure the domain, while "reliability" refers to the precision of the measurement (more measurement error means less reliability).

 © 2021. Thieme. All rights reserved.

(2) Additional confounding domains relevant to the setting of this particular study, or which the study authors identified as important				
**Confounding domain**	**Measured variable(s)**	**Is there evidence that controlling for this variable was unnecessary?[a]**	**Is the confounding domain measured validly and reliably by this variable (or these variables)?**	**Optional: is failure to adjust for this variable (alone) expected to favor the experimental intervention or the comparator?**
			Yes/no/no information	Favor experimental/favor comparator/no information
Gender	No	No	Yes	Both
Race	No	No	Yes	Both
Concomitant pathology	No	No	Yes	Both
Spontaneous intestinal perforation	No	No	Yes	Both
Concomitant medication	No	No	Yes	Both
Concomitant infections	No	No	Yes	Both
Birth weight	Yes	No	Yes	Both (more the comparator >100 g)
Assisted ventilation	Yes	No	Yes	both
Oral feeding	Yes	No	Yes	both
Prenatal exposure to dexamethasone	Yes	No	Yes	Both (more the comparator)
Gestational age	No	No	Yes	No information
Apgar's score at 5 min	Yes	No	Yes	BOTH
Highest level of fraction of inspired oxygen	No	No	Yes	BOTH
Inotropic support	No	No	Yes	BOTH
Resistance bacteria	No	No	Yes	BOTH
DOT/LOT	No	No	Yes	BOTH

[a]In the context of a particular study, variables can be demonstrated not to be confounders and so not included in the analysis: (1) if they are not predictive of the outcome; (2) if they are not predictive of intervention; or (3) because adjustment makes no or minimal difference to the estimated effect of the primary parameter. Note that "no statistically significant association" is not the same as "not predictive."

Preliminary consideration of cointerventions

Complete a row for each important co-intervention (1) listed in the review protocol and (2) relevant to the setting of this particular study, or which the study authors identified as important.

"Important" cointerventions are those for which, in the context of this study, adjustment is expected to lead to a clinically important change in the estimated effect of the intervention

(1) Cointerventions listed in the review protocol		
**Cointervention**	**Is there evidence that controlling for this cointervention was unnecessary (e.g., because it was not administered)?**	**Is presence of this cointervention likely to favor outcomes in the experimental intervention or the comparator**
Surgical intervention	No	Favor experimental/favor comparator/no information
Additional AB treatment	No	Favor experimental/favor comparator/no information
		Favor experimental/favor comparator/no information

 © 2021. Thieme. All rights reserved.

(*Continued*)

		Favor experimental/favor comparator/no information
**(2) Additional cointerventions relevant to the setting of this particular study, or which the study authors identified as important**		
**Cointervention**	**Is there evidence that controlling for this cointervention was unnecessary (e.g., because it was not administered)?**	**Is presence of this cointervention likely to favor outcomes in the experimental intervention or the comparator**
Surgical intervention	No	Both (More the intervention because there are 13 patients who underwent surgery compared with 4 in the comparator group)
Additional AB treatment	No	Favor experimental/favor comparator/no information
		Favor experimental/favor comparator/no information
		Favor experimental/favor comparator/no information

Risk of bias assessment

Responses underlined in green are potential markers for low risk of bias, and responses in red are potential markers for a risk of bias. Where questions relate only to sign posts to other questions, no formatting is used.

Signaling questions	Description	Response options
**Bias due to confounding**		
1.1 Is there potential for confounding of the effect of intervention in this study?<br>**If N/PN to 1.1:** the study can be considered to be at low risk of bias due to confounding and no further signaling questions need be considered		**Y**/PY/PN/N
**If Y/PY to 1.1**: determine whether there is a need to assess time-varying confounding:		
1.2. Was the analysis based on splitting participants' follow up time according to intervention received?<br>**If N/PN**, answer questions relating to baseline confounding (1.4 to 1.6)<br>**If Y/PY**, go to question 1.3.	Not described	**NA**/Y/PY/PN/N/NI
1.3. Were intervention discontinuations or switches likely to be related to factors that are prognostic for the outcome?<br>**If N/PN**, answer questions relating to baseline confounding (1.4 to 1.6)<br>**If Y/PY**, answer questions relating to both baseline and time-varying confounding (1.7 and 1.8)		NA/Y/PY/PN/N/NI
**Questions relating to baseline confounding only**		
1.4. Did the authors use an appropriate analysis method that controlled for all the important confounding domains?	Rates of complications in the two groups were compared by Fisher's exact test (one-sided) or Chi-square analysis. -> NOT SUFFICIENT	NA/Y/PY/PN/**N**/NI
1.5. **If Y/PY to 1.4**: Were confounding domains that were controlled for measured validly and reliably by the variables available in this study?		NA/Y/PY/PN/N/NI
1.6. Did the authors control for any post-intervention variables that could have been affected by the intervention?	BACTERIA IN STOOL AND BLOOD	NA/**Y**/PY/PN/N/NI

(*Continued*)

 © 2021. Thieme. All rights reserved.

(*Continued*)

Signaling questions	Description	Response options
**Questions relating to baseline and time-varying confounding**		
1.7. Did the authors use an appropriate analysis method that controlled for all the important confounding domains and for time-varying confounding?	Rates of complications in the two groups were compared by Fisher's exact test (one-sided) or Chi-square analysis. -> NOT SUFFICIENT	NA/Y/PY/PN/**N**/NI
1.8. **If Y/PY to 1.7**: Were confounding domains that were controlled for measured validly and reliably by the variables available in this study?		NA/Y/PY/PN/N/NI
**Risk of bias judgement**		Low/Moderate/Serious/**Critical**/NI
Optional: What is the predicted direction of bias due to confounding?		Favors experimental/Favors comparator/**Unpredictable**
**Bias in selection of participants into the study**		
2.1. Was selection of participants into the study (or into the analysis) based on participant characteristics observed after the start of intervention?<br>**If N/PN to 2.1:** go to 2.4	2.2 BACTERIAL IN STOOL AND BLOOD	**Y**/PY/PN/N/NI
2.2. **If Y/PY to 2.1**: Were the post-intervention variables that influenced selection likely to be associated with intervention?<br>2.3 **If Y/PY to 2.2**: Were the post-intervention variables that influenced selection likely to be influenced by the outcome or a cause of the outcome?		NA/**Y**/PY/PN/N/NI<br>NA/**Y**/PY/PN/N/NI
2.4. Do start of follow-up and start of intervention coincide for most participants?		Y/PY/PN/N/NI
2.5. **If Y/PY to 2.2 and 2.3, or N/PN to 2.4**: Were adjustment techniques used that are likely to correct for the presence of selection biases?		**NA**/Y/PY/PN/N/NI
**Risk of bias judgement**		Low/Moderate/Serious/**Critical**/NI
Optional: What is the predicted direction of bias due to selection of participants into the study?		Favors experimental/Favors comparator/Toward null/Away from null/**Unpredictable**
**Bias in classification of interventions**		
3.1 Were intervention groups clearly defined?	No, population in the groups were not specified	Y/PY/PN/**N**/NI
3.2 Was the information used to define intervention groups recorded at the start of the intervention?	Yes, AB use and time period	**Y**/PY/PN/N/NI
3.3 Could classification of intervention status have been affected by knowledge of the outcome or risk of the outcome?	There is a protocol	Y/PY/PN/**N**/NI
**Risk of bias judgement**		Low/**Moderate**/Serious/Critical/NI
Optional: What is the predicted direction of bias due to classification of interventions?		Favors experimental/Favors comparator/Toward null/Away from null/**Unpredictable**
**Bias due to deviations from intended interventions**		
**If your aim for this study is to assess the effect of assignment to intervention, answer questions 4.1 and 4.2**		
4.1. Were there deviations from the intended intervention beyond what would be expected in usual practice?	Deviation = additional AB treatment in intervention group	Y/PY/PN/**N**/NI

 © 2021. Thieme. All rights reserved.

(*Continued*)

Signaling questions	Description	Response options
4.2. **If Y/PY to 4.1**: Were these deviations from intended intervention unbalanced between groups *and* likely to have affected the outcome?		NA/Y/PY/PN/N/NI
**If your aim for this study is to assess the effect of starting and adhering to intervention, answer questions 4.3 to 4.6**		
4.3. Were important co-interventions balanced across intervention groups?		**Y/PY**/PN/N/NI
4.4. Was the intervention implemented successfully for most participants?		**Y/PY**/PN/N/NI
4.5. Did study participants adhere to the assigned intervention regimen?		**Y/PY**/PN/N/NI
4.6. **If N/PN to 4.3, 4.4 or 4.5**: Was an appropriate analysis used to estimate the effect of starting and adhering to the intervention?		NA/**Y/PY**/PN/N/NI
**Risk of bias judgement**		**Low**/Moderate/Serious/Critical/NI
Optional: What is the predicted direction of bias due to deviations from the intended interventions?		Favors experimental/Favors comparator/Toward null/Away from null/**Unpredictable**
**Bias due to missing data**		
5.1 Were outcome data available for all, or nearly all, participants?	Yes, but Stool samples were obtained for culture at onset in 47 cases out of 90 and impact of antibiotic treatment on the faecal flora could be measured in 27 patients	Y/PY/**PN**/N/NI
5.2 Were participants excluded due to missing data on intervention status?	AB treatment data were complete	Y/PY/PN/**N**/NI
5.3 Were participants excluded due to missing data on other variables needed for the analysis?	Yes,<br>Stool samples were obtained for culture at onset in 47 cases out of 90 and impact of antibiotic treatment on the faecal flora could be measured in 27 patients	**Y**/PY/PN/N/NI
5.4 **If PN/N to 5.1, or Y/PY to 5.2 or 5.3**: Are the proportion of participants and reasons for missing data similar across interventions?		NA/Y/PY/**PN**/N/NI
5.5 **If PN/N to 5.1, or Y/PY to 5.2 or 5.3**: Is there evidence that results were robust to the presence of missing data?		**NA**/Y/PY/PN/N/NI
**Risk of bias judgement**		Low/Moderate/**Serious**/Critical/NI
Optional: What is the predicted direction of bias due to missing data?		Favors experimental/Favors comparator/**Toward null**/Away from null/Unpredictable
**Bias in measurement of outcomes**		
6.1 Could the outcome measure have been influenced by knowledge of the intervention received?		Y/PY/PN/**N**/NI
6.2 Were outcome assessors aware of the intervention received by study participants?		**Y**/PY/PN/N/NI
6.3 Were the methods of outcome assessment comparable across intervention groups?		Y/**PY**/PN/N/NI
6.4 Were any systematic errors in measurement of the outcome related to intervention received?		Y/PY/**PN**/N/NI

(*Continued*)

 © 2021. Thieme. All rights reserved.

(*Continued*)

Signaling questions	Description	Response options
**Risk of bias judgement**		**Low**/Moderate/Serious/Critical/NI
Optional: What is the predicted direction of bias due to measurement of outcomes?		Favors experimental/Favors comparator/Toward null/Away from null/ **Unpredictable**
	**Bias in selection of the reported result**	
Is the reported effect estimate likely to be selected, on the basis of the results, from ...		
7.1. ... multiple outcome measurements within the outcome domain?	peritonitis—redness of at least one-quarter of the anterior abdominal wall, present for more than 24 hour, in association with local tenderness and generalized abdominal distension or the presence of purulent exudate within the abdomen; intestinal perforation—presence of free air within the peritoneal cavity on abdominal radiographs or a hole in the bowel on direct examination;	**Y**/PY/PN/N/NI
7.2 ... Multiple *analyses* of the intervention-outcome relationship?		Y/PY/**PN**/N/NI
7.3 ... Different subgroups?	>2,200 g, <2,200 g	**Y** PY/PN/N/NI
**Risk of bias judgement**		Low/Moderate/ **Serious**/Critical/NI
Optional: What is the predicted direction of bias due to selection of the reported result?		Favors experimental/Favors comparator/Toward null/Away from null/ **Unpredictable**
	**Overall bias**	
**Risk of bias judgement**		Low / Moderate/ **Serious**/Critical/NI
Optional: What is the overall predicted direction of bias for this outcome?		Favors experimental/Favors comparator/Toward null /Away from null/ **Unpredictable**

ROBINS-I tool: anaerobic antimicrobial therapy after necrotizing enterocolitis in VLBW infants (Autmizguine et al[25])

Specify a target randomized trial specific to the study

Design	Individually randomized/cluster randomized/matched (e.g., crossover)
Participants	1,390 infants
Experimental intervention	Anaerobic antimicrobial therapy
Comparator	Infants who were not exposed anaerobic antimicrobial therapy

Is your aim for this study ...?

✓	To assess the effect of assignment to intervention
☐	To assess the effect of starting and adhering to intervention

 © 2021. Thieme. All rights reserved.

Specify the outcome

Specify which outcome is being assessed for risk of bias (typically from among those earmarked for the Summary of Findings table). Specify whether this is a proposed benefit or harm of intervention.

Case population definition (inclusion exclusion criteria), aim, collection of data, study design, statistical analysis

Specify the numerical result being assessed

In case of multiple alternative analyses being presented, specify the numeric result (e.g., RR = 1.52 [95% CI: 0.83–2.77]) and/or a reference (e.g., to a table, figure or paragraph) that uniquely defines the result being assessed.

Preliminary consideration of confounders

Complete a row for each important confounding domain (i) listed in the review protocol; and (ii) relevant to the setting of this particular study, or which the study authors identified as potentially important.

"Important" confounding domains are those for which, in the context of this study, adjustment is expected to lead to a clinically important change in the estimated effect of the intervention

(1) Confounding domains listed in the review protocol				
**Confounding domain**	**Measured variable(s)**	**Is there evidence that controlling for this variable was unnecessary?[a]**	**Is the confounding domain measured validly and reliably by this variable (or these variables)?**	**Optional: is failure to adjust for this variable (alone) expected to favor the experimental intervention or the comparator?**
			yes/no/no information	Favor experimental/favor comparator/no information
Concomitant pathology				
Concomitant spontaneous intestinal perforation				
Concomitant medication				
Concomitant infections				
Birth weight				
Assisted ventilation				
Oral feeding				
Prenatal exposure to dexamethasone				
Gestational age				
Apgar's score at 5 min				
Highest level of fraction of inspired oxygen				
Inotropic support				

Note: "Validity" refers to whether the confounding variable or variables fully measure the domain, while "reliability" refers to the precision of the measurement (more measurement error means less reliability).

 © 2021. Thieme. All rights reserved.

(2) Additional confounding domains relevant to the setting of this particular study, or which the study authors identified as important				
Confounding domain	Measured variable(s)	Is there evidence that controlling for this variable was unnecessary?[a]	Is the confounding domain measured validly and reliably by this variable (or these variables)?	Optional: is failure to adjust for this variable (alone) expected to favor the experimental intervention or the comparator?
			Yes/no/no information	Favor experimental/favor comparator/no information
Gender	Yes	No	Yes	Both
Race	Yes	No	Yes	Both
Concomitant pathology	No	No	Yes	Both
Spontaneous intestinal perforation	No	No	Yes	Both
Concomitant medication	No	No	Yes	Both
Concomitant infections	No	No	Yes	Both
Birth weight	Yes	No	Yes	Both
Assisted ventilation	Yes	No	Yes	Both
Oral feeding	Yes	No	Yes	Both
Prenatal exposure to dexamethasone	Yes	No	Yes	Both
Gestational age	Yes	No	Yes	Both
Apgar's score at 5 min	Yes	No	Yes	Both
Highest level of fraction of inspired oxygen	Yes	No	Yes	Both
Inotropic support	Yes	No	Yes	Both
Resistance bacteria	No	No	Yes	Both
DOT/LOT	No	No	Yes	Both

[a]In the context of a particular study, variables can be demonstrated not to be confounders and so not included in the analysis: (a) if they are not predictive of the outcome; (b) if they are not predictive of intervention; or (c) because adjustment makes no or minimal difference to the estimated effect of the primary parameter. Note that "no statistically significant association" is not the same as "not predictive."

Preliminary consideration of cointerventions

Complete a row for each important co-intervention (i) listed in the review protocol; and (ii) relevant to the setting of this particular study, or which the study authors identified as important.

"Important" cointerventions are those for which, in the context of this study, adjustment is expected to lead to a clinically important change in the estimated effect of the intervention

(1) Cointerventions listed in the review protocol		
Cointervention	Is there evidence that controlling for this cointervention was unnecessary (e.g., because it was not administered)?	Is presence of this cointervention likely to favor outcomes in the experimental intervention or the comparator
Surgical intervention	No	Favor experimental/favor comparator/no information
Additional antibiotic treatment	No	

 © 2021. Thieme. All rights reserved.

(*Continued*)

		Favor experimental /favor comparator/no information
**(2) Additional cointerventions relevant to the setting of this particular study, or which the study authors identified as important**		
**Cointervention**	**Is there evidence that controlling for this cointervention was unnecessary (e.g., because it was not administered)?**	**Is presence of this cointervention likely to favor outcomes in the experimental intervention or the comparator**
Surgical intervention	Maybe (the study is a matched cohort)	Favor experimental/favor comparator/no information
Additional ab treatment	Maybe (the study is a matched cohort)	Favor experimental/favor comparator/no information
		Favor experimental/favor comparator/no information
		Favor experimental/favor comparator/no information

Risk of bias assessment

Responses underlined in green are potential markers for low risk of bias, and responses in red are potential markers for a risk of bias. Where questions relate only to sign posts to other questions, no formatting is used.

Signaling questions	Description	Response options
	**Bias due to confounding**	
1.1 Is there potential for confounding of the effect of intervention in this study?<br>**If N/PN to 1.1:** the study can be considered to be at low risk of bias due to confounding and no further signaling questions need be considered	Resistance bacteria<br>Concomitant pathologies<br>Concomitant medication<br>Spontaneous intestinal perforation<br>NEC severity	**Y**/PY/PN/N
**If Y/PY to 1.1**: determine whether there is a need to assess time-varying confounding:		
1.2. Was the analysis based on splitting participants' follow up time according to intervention received?<br>**If N/PN**, answer questions relating to baseline confounding (1.4 to 1.6)<br>**If Y/PY**, go to question 1.3.	Not described	**NA**/Y/PY/PN/N/NI
1.3. Were intervention discontinuations or switches likely to be related to factors that are prognostic for the outcome?<br>**If N/PN**, answer questions relating to baseline confounding (1.4 to 1.6)<br>**If Y/PY**, answer questions relating to both baseline and time-varying confounding (1.7 and 1.8)		NA/**Y**/PY/PN/N/NI
**Questions relating to baseline confounding only**		
1.4. Did the authors use an appropriate analysis method that controlled for all the important confounding domains?	y, propensity score (PS) 1:1 matching was used to 118 AUTMIZGUINE et al ensure comparison of similar infants	NA/**Y**/PY/PN/N/NI
1.5. **If Y/PY to 1.4**: Were confounding domains that were controlled for measured validly and reliably by the variables available in this study?		NA/**Y**/PY/PN/N/NI
		NA/**Y**/PY/PN/N/NI

(*Continued*)

 © 2021. Thieme. All rights reserved.

(*Continued*)

Signaling questions	Description	Response options
1.6. Did the authors control for any post-intervention variables that could have been affected by the intervention?	composite of progression from medical to surgical NEC or death	
**Questions relating to baseline and time-varying confounding**		
1.7. Did the authors use an appropriate analysis method that controlled for all the important confounding domains and for time-varying confounding?	y, propensity score (PS) 1:1 matching was used to 118 AUTMIZGUINE et al ensure comparison of similar infants	NA/**Y**/PY/PN/N/NI
1.8. **If Y/PY to 1.7**: Were confounding domains that were controlled for measured validly and reliably by the variables available in this study?		NA/**Y**/PY/PN/N/NI
**Risk of bias judgement**		Low/**Moderate**/Serious/Critical/NI
Optional: What is the predicted direction of bias due to confounding?		Favors experimental/Favors comparator/**Unpredictable**
**Bias in selection of participants into the study**		
2.1. Was selection of participants into the study (or into the analysis) based on participant characteristics observed after the start of intervention?<br>**If N/PN to 2.1:** go to 2.4		Y/PY/**PN**/N/NI
2.2. **If Y/PY to 2.1**: Were the post-intervention variables that influenced selection likely to be associated with intervention?<br>2.3 **If Y/PY to 2.2**: Were the post-intervention variables that influenced selection likely to be influenced by the outcome or a cause of the outcome?		NA/Y/PY/**PN**/N/NI<br>NA/Y/PY/**PN**/N/NI
2.4. Do start of follow-up and start of intervention coincide for most participants?		Y/**PY**/PN/N/NI
2.5. **If Y/PY to 2.2 and 2.3, or N/PN to 2.4**: Were adjustment techniques used that are likely to correct for the presence of selection biases?		NA/Y/PY/PN/N/NI
**Risk of bias judgement**		**Low**/Moderate/Serious/Critical/NI
Optional: What is the predicted direction of bias due to selection of participants into the study?		Favors experimental/Favors comparator/Toward null/Away from null/**Unpredictable**
**Bias in classification of interventions**		
3.1 Were intervention groups clearly defined?	The diagnosis and severity of NEC were assigned at each site by the attending neonatologist and included either medical NEC or surgical NEC. The assessment of NEC severity was not standardized	Y/PY/PN/**N**/NI
3.2 Was the information used to define intervention groups recorded at the start of the intervention?	Yes, AB use and time period	**Y**/PY/PN/N/NI
3.3 Could classification of intervention status have been affected by knowledge of the outcome or risk of the outcome?	There is a protocol	Y/**PY**/PN/N/NI
**Risk of bias judgement**		Low/Moderate/**Serious** / Critical/NI
Optional: What is the predicted direction of bias due to classification of interventions?		Favors experimental/Favors comparator/Toward null/

 © 2021. Thieme. All rights reserved.

(*Continued*)

Signaling questions	Description	Response options
		Away from null/ **Unpredictable**
**Bias due to deviations from intended interventions**		
**If your aim for this study is to assess the effect of assignment to intervention, answer questions 4.1 and 4.2**		
4.1. Were there deviations from the intended intervention beyond what would be expected in usual practice?	Deviation = additional AB treatment in intervention group	Y/PY/<u>PN/**N**</u>/NI
4.2. **If Y/PY to 4.1**: Were these deviations from intended intervention unbalanced between groups *and* likely to have affected the outcome?		NA/Y/PY/<u>PN/N</u>/NI
**If your aim for this study is to assess the effect of starting and adhering to intervention, answer questions 4.3 to 4.6**		
4.3. Were important co-interventions balanced across intervention groups?		<u>Y/PY</u>/PN/N/NI
4.4. Was the intervention implemented successfully for most participants?		<u>Y/PY</u>/PN/N/NI
4.5. Did study participants adhere to the assigned intervention regimen?		<u>Y/PY</u>/PN/N/NI
4.6. **If N/PN to 4.3, 4.4 or 4.5**: Was an appropriate analysis used to estimate the effect of starting and adhering to the intervention?		NA/<u>Y/PY</u>/PN/N/NI
**Risk of bias judgement**		**Low** / Moderate/Serious / Critical/NI
Optional: What is the predicted direction of bias due to deviations from the intended interventions?		Favors experimental/Favors comparator/Toward null/Away from null/ **Unpredictable**
**Bias due to missing data**		
5.1 Were outcome data available for all, or nearly all, participants?	Yes,	<u>Y/**PY**</u>/PN/N/NI
5.2 Were participants excluded due to missing data on intervention status?	AB treatment data were complete	Y/PY/<u>PN/**N**</u>/NI
5.3 Were participants excluded due to missing data on other variables needed for the analysis?	Yes,	Y/PY/<u>PN/**N**</u>/NI
5.4 **If PN/N to 5.1, or Y/PY to 5.2 or 5.3**: Are the proportion of participants and reasons for missing data similar across interventions?		NA/<u>Y/PY</u>/PN/N/NI
5.5 **If PN/N to 5.1, or Y/PY to 5.2 or 5.3**: Is there evidence that results were robust to the presence of missing data?		NA/<u>Y/PY</u>/PN/N/NI
**Risk of bias judgement**		**Low**/Moderate/Serious / Critical/NI
Optional: What is the predicted direction of bias due to missing data?		Favors experimental/Favors comparator/**Toward null**/ Away from null/Unpredictable
**Bias in measurement of outcomes**		
6.1 Could the outcome measure have been influenced by knowledge of the intervention received?		Y/PY/<u>PN/**N**</u>/NI
6.2 Were outcome assessors aware of the intervention received by study participants?		**Y**/PY/<u>PN/N</u>/NI
6.3 Were the methods of outcome assessment comparable across intervention groups?		<u>Y/**PY**</u>/PN/N/NI

(*Continued*)

 © 2021. Thieme. All rights reserved.

(*Continued*)

Signaling questions	Description	Response options
6.4 Were any systematic errors in measurement of the outcome related to intervention received?		Y/PY/**PN**/N/NI
**Risk of bias judgement**		Low/**Moderate**/Serious/Critical/NI
Optional: What is the predicted direction of bias due to measurement of outcomes?		Favors experimental/Favors comparator/Toward null/Away from null/**Unpredictable**
**Bias in selection of the reported result**		
Is the reported effect estimate likely to be selected, on the basis of the results, from ...		
7.1. ... multiple outcome *measurements* within the outcome domain?		Y/PY/PN/**N**/NI
7.2 ... multiple *analyses* of the intervention-outcome relationship?		Y/PY/PN/**N**/NI
7.3 ... different subgroups?		Y/PY/PN/**N**/NI
**Risk of bias judgement**		**Low**/Moderate/Serious/Critical/NI
Optional: What is the predicted direction of bias due to selection of the reported result?		Favors experimental/Favors comparator/Toward null/Away from null/**Unpredictable**
**Overall bias**		
**Risk of bias judgement**		**Low**/Moderate/Serious/Critical/NI
Optional: What is the overall predicted direction of bias for this outcome?		Favors experimental/Favors comparator/Toward null/Away from null/**Unpredictable**

ROBINS-I tool (stage II): for each study—broad-spectrum antibiotic plus metronidazole may not prevent the deterioration of necrotizing enterocolitis from stage II to III in full-term and near-term infants (Luo et al[26])

Specify a target randomized trial specific to the study

Design	Individually randomized/cluster randomized/matched (e.g., crossover) nonrandomized
Participants	229 infants
Experimental intervention	Broad-spectrum antibiotic therapy + metronidazole
Comparator	Broad-spectrum antibiotic therapy

Is your aim for this study ...?

✓	To assess the effect of assignment to intervention
☐	To assess the effect of starting and adhering to intervention

 © 2021. Thieme. All rights reserved.

Specify the outcome

Specify which outcome is being assessed for risk of bias (typically from among those earmarked for the Summary of Findings table). Specify whether this is a proposed benefit or harm of intervention.

Case population definition (inclusion exclusion criteria), aim, collection of data, study design, statistical analysis

Specify the numerical result being assessed

In case of multiple alternative analyses being presented, specify the numeric result (e.g., RR = 1.52 [95% CI 0.83–2.77]) and/or a reference (e.g., to a table, figure or paragraph) that uniquely defines the result being assessed.

Preliminary consideration of confounders

Complete a row for each important confounding domain (i) listed in the review protocol; and (ii) relevant to the setting of this particular study, or which the study authors identified as potentially important.

"Important" confounding domains are those for which, in the context of this study, adjustment is expected to lead to a clinically important change in the estimated effect of the intervention

**(1) Confounding domains listed in the review protocol**				
**Confounding domain**	**Measured variable(s)**	**Is there evidence that controlling for this variable was unnecessary?**[a]	**Is the confounding domain measured validly and reliably by this variable (or these variables)?**	**Optional: is failure to adjust for this variable (alone) expected to favor the experimental intervention or the comparator?**
			Yes/no/no information	Favor experimental/favor comparator/no information
Concomitant pathology				
Concomitant spontaneous intestinal perforation				
Concomitant medication				
Concomitant infections				
Birth weight				
Assisted ventilation				
Oral feeding				
Prenatal exposure to dexamethasone				
Gestational age				
Apgar's score at 5 min				
Highest level of fraction of inspired oxygen				
Inotropic support				

Note: "Validity" refers to whether the confounding variable or variables fully measure the domain, while "reliability" refers to the precision of the measurement (more measurement error means less reliability).

 © 2021. Thieme. All rights reserved.

(2) Additional confounding domains relevant to the setting of this particular study, or which the study authors identified as important				
**Confounding domain**	**Measured variable(s)**	**Is there evidence that controlling for this variable was unnecessary?[a]**	**Is the confounding domain measured validly and reliably by this variable (or these variables)?**	**Optional: is failure to adjust for this variable (alone) expected to favor the experimental intervention or the comparator?**
			Yes/no/no information	Favor experimental/favor comparator/no information
Gender	Yes	No	Yes	Both
Race	No	No	Yes	Both
Concomitant pathology	No	No	Yes	Both
Spontaneous intestinal perforation	Yes	No	Yes	Both
Concomitant medication	No	No	Yes	Both
Concomitant infections	No	No	Yes	Both
Birth weight	Yes	No	Yes	Both
Assisted ventilation	No	No	Yes	Both
Oral feeding	Yes	No	Yes	Both
Prenatal exposure to dexamethasone	No	No	Yes	Both
Gestational age	Yes	No	Yes	No information
Apgar SCORE AT 5 MIN	No	No	Yes	Both
Highest level of fraction of inspired oxygen	No	No	Yes	Both
Inotropic support	No	No	Yes	Both
Resistance bacteria	No	No	Yes	Both
DOT/LOT	Yes	No	Yes	Both

[a]In the context of a particular study, variables can be demonstrated not to be confounders and so not included in the analysis: (1) if they are not predictive of the outcome; (2) if they are not predictive of intervention; or (3) because adjustment makes no or minimal difference to the estimated effect of the primary parameter. Note that "no statistically significant association" is not the same as "not predictive."

Preliminary consideration of cointerventions

Complete a row for each important co-intervention (1) listed in the review protocol and (2) relevant to the setting of this particular study, or which the study authors identified as important.

"Important" cointerventions are those for which, in the context of this study, adjustment is expected to lead to a clinically important change in the estimated effect of the intervention.

(1) Cointerventions listed in the review protocol		
**Cointervention**	**Is there evidence that controlling for this cointervention was unnecessary (e.g., because it was not administered)?**	**Is presence of this cointervention likely to favor outcomes in the experimental intervention or the comparator**
Surgical intervention	No	Favor experimental/favor comparator/no information
Additional ab treatment	No	Favor experimental/favor comparator/no information

 © 2021. Thieme. All rights reserved.

(*Continued*)

		Favor experimental/favor comparator/no information
		Favor experimental/favor comparator/no information
**(2) Additional cointerventions relevant to the setting of this particular study, or which the study authors identified as important**		
**Cointervention**	**Is there evidence that controlling for this cointervention was unnecessary (e.g., because it was not administered)?**	**Is presence of this cointervention likely to favor outcomes in the experimental intervention or the comparator**
Surgical intervention	No	It is not clear if the data regarding stage 3, they did not show data regarding surgical intervention.
Additional ab treatment	No	The outcome depends also by the therapy, not just on the metronidazole addition and it is not explained who received what.
		Favor experimental/favor comparator/no information
		Favor experimental/favor comparator/no information

Risk of bias assessment

Signaling questions	Description	Response options
**Bias due to confounding**		
1.1 Is there potential for confounding of the effect of intervention in this study?<br>**If N/PN to 1.1:** the study can be considered to be at low risk of bias due to confounding and no further signaling questions need be considered		**Y**/PY/PN/
**If Y/PY to 1.1**: determine whether there is a need to assess time-varying confounding:		
1.2. Was the analysis based on splitting participants' follow up time according to intervention received?<br>**If N/PN**, answer questions relating to baseline confounding (1.4 to 1.6)<br>**If Y/PY**, go to question 1.3.	No, it was not described how the participant were followed up	**NA**/Y/PY/PN/N/NI
1.3. Were intervention discontinuations or switches likely to be related to factors that are prognostic for the outcome?<br>**If N/PN**, answer questions relating to baseline confounding (1.4 to 1.6)<br>**If Y/PY**, answer questions relating to both baseline and time-varying confounding (1.7 and 1.8)		NA/Y/PY/PN/N/NI
**Questions relating to baseline confounding only**		
1.4. Did the authors use an appropriate analysis method that controlled for all the important confounding domains?	Rates of complications in the two groups were compared by Fisher's exact test (one-sided) or Mann- Whitney. They adjust for propensity scores as well, but they did	NA/**Y**/PY/PN/N/NI

(*Continued*)

 © 2021. Thieme. All rights reserved.

(*Continued*)

Signaling questions	Description	Response options
	not considered lots of variables.	
1.5. **If Y/PY to 1.4**: Were confounding domains that were controlled for measured validly and reliably by the variables available in this study?		NA/Y/PY/**PN**/N/NI
1.6. Did the authors control for any post-intervention variables that could have been affected by the intervention?		**NA**/Y/PY/PN/N/NI
**Questions relating to baseline and time-varying confounding**		
1.7. Did the authors use an appropriate analysis method that controlled for all the important confounding domains and for time-varying confounding?		NA/Y/PY/PN/N/NI
1.8. **If Y/PY to 1.7**: Were confounding domains that were controlled for measured validly and reliably by the variables available in this study?		NA/Y/PY/PN/N/NI
**Risk of bias judgement**		Low/Moderate/ **Serious**/Critical/NI
Optional: What is the predicted direction of bias due to confounding?		Favors experimental/Favors comparator/**Unpredictable**
**Bias in selection of participants into the study**		
2.1. Was selection of participants into the study (or into the analysis) based on participant characteristics observed after the start of intervention?<br>**If N/PN to 2.1:** go to 2.4		Y/PY/**PN**/N/NI
2.2. **If Y/PY to 2.1**: Were the post-intervention variables that influenced selection likely to be associated with intervention?<br>2.3 **If Y/PY to 2.2**: Were the post-intervention variables that influenced selection likely to be influenced by the outcome or a cause of the outcome?		NA/Y/PY/PN/N/NI<br>NA/Y/PY/PN/N/NI
2.4. Do start of follow-up and start of intervention coincide for most participants?		Y/**PY**/PN/N/NI
2.5. **If Y/PY to 2.2 and 2.3, or N/PN to 2.4**: Were adjustment techniques used that are likely to correct for the presence of selection biases?		NA/Y/PY/PN/N/NI
**Risk of bias judgement**		**Low**/Moderate/Serious/Critical/NI
Optional: What is the predicted direction of bias due to selection of participants into the study?		Favors experimental/Favors comparator/Toward null/Away from null/ **Unpredictable**
**Bias in classification of interventions**		
3.1 Were intervention groups clearly defined?		Y/PY/**PN**/N/NI
3.2 Was the information used to define intervention groups recorded at the start of the intervention?	Yes, AB use and time period	**Y**/PY/PN/N/NI
3.3 Could classification of intervention status have been affected by knowledge of the outcome or risk of the outcome?	There was no protocol	**Y**/PY/PN/N/NI
**Risk of bias judgement**		Low/**Moderate**/Serious/Critical/NI
Optional: What is the predicted direction of bias due to classification of interventions?		Favors experimental/Favors comparator/Toward null/Away from null/ **Unpredictable**

 © 2021. Thieme. All rights reserved.

(*Continued*)

Signaling questions	Description	Response options
**Bias due to deviations from intended interventions**		
**If your aim for this study is to assess the effect of assignment to intervention, answer questions 4.1 and 4.2**		
4.1. Were there deviations from the intended intervention beyond what would be expected in usual practice?		Y/PY/PN/**N**/NI
4.2. **If Y/PY to 4.1**: Were these deviations from intended intervention unbalanced between groups *and* likely to have affected the outcome?		NA/Y/PY/PN/N/NI
**If your aim for this study is to assess the effect of starting and adhering to intervention, answer questions 4.3 to 4.6**		
4.3. Were important co-interventions balanced across intervention groups?		Y/PY/PN/N/NI
4.4. Was the intervention implemented successfully for most participants?		Y/PY/PN/N/NI
4.5. Did study participants adhere to the assigned intervention regimen?		Y/PY/PN/N/NI
4.6. **If N/PN to 4.3, 4.4 or 4.5**: Was an appropriate analysis used to estimate the effect of starting and adhering to the intervention?		NA/Y/PY/PN/N/NI
**Risk of bias judgement**		**Low**/Moderate/Serious/Critical/NI
Optional: What is the predicted direction of bias due to deviations from the intended interventions?		Favors experimental/Favors comparator/Toward null/Away from null/**Unpredictable**
**Bias due to missing data**		
5.1 Were outcome data available for all, or nearly all, participants?		Y/PY/**PN**/N/NI
5.2 Were participants excluded due to missing data on intervention status?		Y/PY/PN/**N**/NI
5.3 Were participants excluded due to missing data on other variables needed for the analysis?		**Y**/PY/PN/N/NI
5.4 **If PN/N to 5.1, or Y/PY to 5.2 or 5.3**: Are the proportion of participants and reasons for missing data similar across interventions?		NA/Y/**PY**/PN/N/NI
5.5 **If PN/N to 5.1, or Y/PY to 5.2 or 5.3**: Is there evidence that results were robust to the presence of missing data?		**NA**/Y/PY/PN/N/NI
**Risk of bias judgement**		Low/**Moderate**/Serious/Critical/NI
Optional: What is the predicted direction of bias due to missing data?		Favors experimental/Favors comparator/**Toward null**/Away from null/Unpredictable
**Bias in measurement of outcomes**		
6.1 Could the outcome measure have been influenced by knowledge of the intervention received?		Y/PY/PN/**N**/NI
6.2 Were outcome assessors aware of the intervention received by study participants?		**Y**/PY/PN/N/NI
6.3 Were the methods of outcome assessment comparable across intervention groups?		Y/**PY**/PN/N/NI
6.4 Were any systematic errors in measurement of the outcome related to intervention received?		Y/PY/**PN**/N/NI
**Risk of bias judgement**		**Low**/Moderate/Serious/Critical/NI

(*Continued*)

 © 2021. Thieme. All rights reserved.

(*Continued*)

Signaling questions	Description	Response options
Optional: What is the predicted direction of bias due to measurement of outcomes?		Favors experimental/Favors comparator/Toward null/Away from null/**Unpredictable**
	**Bias in selection of the reported result**	
Is the reported effect estimate likely to be selected, on the basis of the results, from ...		
7.1. ... multiple outcome *measurements* within the outcome domain?		Y/**PY**/PN/N/NI
7.2 ... multiple *analyses* of the intervention-outcome relationship?		Y/PY/**PN**/N/NI
7.3 ... different *subgroups*?		**Y**/PY/PN/N/NI
**Risk of bias judgement**		Low/Moderate/**Serious**/Critical/NI
Optional: What is the predicted direction of bias due to selection of the reported result?		Favors experimental/Favors comparator/Toward null/Away from null/**Unpredictable**
	**Overall bias**	
**Risk of bias judgement**		Low/Moderate/**Serious**/Critical/NI
Optional: What is the overall predicted direction of bias for this outcome?		Favors experimental/Favors comparator/Toward null/Away from null/**Unpredictable**

 © 2021. Thieme. All rights reserved.
